# Balancing control and autonomy in master surgery scheduling: Benefits of ICU quotas for recovery units

**DOI:** 10.1007/s10729-021-09588-8

**Published:** 2022-02-09

**Authors:** Steffen Heider, Jan Schoenfelder, Thomas Koperna, Jens O. Brunner

**Affiliations:** 1 Faculty of Business and Economics, University of Augsburg, Universitätsstraße 16, 86159 Augsburg, Germany; 2 Unit of Digitalization and Business Analytics, Universitätsklinikum Augsburg, Stenglinstraße 2, 86156 Augsburg, Germany; 3 Department of Operating Room Management, Universitätsklinikum Augsburg, Stenglinstraße 2, 86156 Augsburg, Germany

**Keywords:** Healthcare, Master surgery schedule, Operating theater, Downstream, Operations research, Operations management

## Abstract

When scheduling surgeries in the operating theater, not only the resources within the operating theater have to be considered but also those in downstream units, e.g., the intensive care unit and regular bed wards of each medical specialty. We present an extension to the master surgery schedule, where the capacity for surgeries on ICU patients is controlled by introducing downstream-dependent block types – one for both ICU and ward patients and one where surgeries on ICU patients must not be performed. The goal is to provide better control over post-surgery patient flows through the hospital while preserving each medical specialty’s autonomy over its operational surgery scheduling. We propose a mixed-integer program to determine the allocation of the new block types within either a given or a new master surgery schedule to minimize the maximum workload in downstream units. Using a simulation model supported by seven years of data from the University Hospital Augsburg, we show that the maximum workload in the intensive care unit can be reduced by up to 11.22% with our approach while maintaining the existing master surgery schedule. We also show that our approach can achieve up to 79.85% of the maximum workload reduction in the intensive care unit that would result from a fully centralized approach. We analyze various hospital setting instances to show the generalizability of our results. Furthermore, we provide insights and data analysis from the implementation of a quota system at the University Hospital Augsburg.

**Supplementary Information:**

The online version contains supplementary material available at 10.1007/s10729-021-09588-8.

## Highlights

- We introduce ICU quotas for the master surgery schedule
- We focus on the tactical level to reduce peak workload on the recovery units, especially the ICU
- We use a combined optimization and simulation approach to analyze the schedules
- We show that peak workload reduction is possible without changing the master surgery schedule
- We report on the implementation of ICU quotas at our cooperating hospital

## Introduction

Scheduling surgeries in the operating theater (OT) is a challenging task as a multitude of resources, e.g., physicians, nurses, anesthetists, operating rooms (OR), and equipment have to be considered. Additionally, resources of downstream units, e.g., bed capacity in the intensive care unit (ICU), the intermediate care unit (IMC), and in the regular bed wards of each medical specialty, are limited and need to be incorporated in the planning process. In practice, a master surgery schedule (MSS) is often used on the tactical level to provide planning certainty for all medical specialties and to reduce the complexity of daily surgery scheduling on the operational level. In an MSS, OT capacity is assigned to different specialties, in which each specialty is assigned some number of OR blocks each day. This allows each specialty to schedule its patients individually in their given OR blocks. The reduced complexity allows specialties to react to unforeseen events, e.g., emergency cases or deviations from the scheduled surgery time of patients, autonomously, but other problems may arise. For example, the decentralized planning of surgeries may cause significant daily fluctuations in the ICU, a downstream unit of the OT shared by all specialties, regarding the number of admissions, the daily workload, and the number of discharges. On some days, the number of admissions can be higher than the number of planned discharges leading to early discharges, which harm patients’ recovery [3]. On other days, the number of admissions can be lower than the planned number of discharges, which leads to idle capacity in the ICU. Additional scheduling rules are necessary to limit the daily number of scheduled patients for each downstream unit in the OT. In practice, these restrictions are mostly considered on an operational level but not on the tactical level. The lack of an integrated planning approach on the tactical level leads to increased planning efforts on the operational level, especially in the ICU.

In this paper, we present an extension to the MSS using distinct block types for individual downstream units to control downstream resource consumption on a tactical level. We develop a mixed-integer program (MIP) to minimize the maximum workload for nurses and physicians in downstream units using patient-to-nurse ratios as well as the workload for admissions, daily routines, and discharges. The model can be used to calculate a new MSS where ICU blocks (allowing surgeries on ICU and ward patients) and ward blocks (where ICU patients cannot be treated) of each specialty have to be allocated to rooms and days using capacity restrictions derived from strategic planning. Our approach can also be applied to an existing MSS to decide whether the given blocks should be declared ICU blocks or ward blocks. Maintaining an existing MSS is usually preferred by hospitals due to the major process disruptions that derive from changing the MSS. We evaluate the performance in terms of the maximum workload in each downstream unit of an existing MSS and a newly calculated MSS using a simulation model for both the traditional approach and our extension. We use OT and ward data from 2010 through 2016 from the University Hospital Augsburg (UKA), a tertiary care hospital in Germany with more than 1,700 beds. We used a modified version of our model to calculate ICU quotas for the OT of UKA which were implemented in practice in January 2020. We describe the implementation process and measure the impact the new process has in practice by comparing OT and ICU data from two two-month periods, one in 2019, where no quota system had been implemented, and one in 2020, where the quota system had been implemented.

Our contributions are the following: First, in the case study of UKA, we show that our approach outperforms the traditional approach, where no downstream-related surgery capacity is allocated in the OT, by up to 11.22% in the ICU. Second, we also show that a new MSS might not be necessary since our approach can realize most of the peak workload reduction in the ICU with an existing MSS. Third, our approach can yield up to 79.85% of potential maximum workload reduction compared to a central planning approach, scheduling surgeries considering downstream workload leveling on the operational level. Hence, our approach reaps most of the potential benefits while maintaining most of the patient scheduling autonomy of each specialty. Fourth, based on LOS data from UKA, we built 64 additional instances, ranging from small OTs with just two rooms up to large OTs with 16 rooms, where we show that the results from UKA apply in general. Fifth, based on our results, an adapted version of our approach was implemented at UKA in January 2020. The actual implementation is a major advantage over other MSS optimization approaches previously presented in the literature. We provide insights, lessons learned, and present OT and ICU data demonstrating the real-world effects of an ICU quota system from the practical implementation at UKA.

The remainder of the paper is structured as follows. In Section 2, the relevant literature is reviewed. In Section 3, we present the methodology of our approach. The results of the numerical study are discussed in Section 4. In Section 5, we present managerial insights from the implementation at UKA. Section 6 concludes our work.

## Literature Overview

There has been a lot of research interest in surgery scheduling in the operations research/management science community. Several literature reviews with a focus on the OT exist. For a broad review, see Cardoen et al. [11] and Samudra et al. [28] who include work up until 2009 and 2016, respectively. For more recent reviews, see Zhu et al. [40] and Gür and Eren [18]. For a more specific overview focused on multiple departments including the OT, see Vanberkel et al. [37] and Guerriero and Guido [17].

Following the framework of Cardoen et al. [11], the OT literature can be divided into three planning levels: strategic, tactical, and operational. Planning on the strategic level mostly consists of problems focused on case mix planning, where OT time is divided between specialties for surgical groups to maximize revenue. For an overview of the case mix planning literature, see Hof et al. [20]. On the tactical level, the results from case mix planning are used and are distributed either in a block plan (also called MSS), an open scheduling approach, or a mix of both called modified block plan. The main feature of tactical plans, however, is the cyclic approach that the developed plans are repeated every one to four weeks. On the operational level, individual patients are then scheduled and sequenced according to the tactical plan determined in the previous stage. The overview in the following is solely focused on the literature on the tactical level or a combination of the tactical and operational level with at least one downstream unit. Literature with a sole focus on operational surgery scheduling – both advance and allocation scheduling – is not included. Here, we refer to Shehadeh and Padman [32] who review the literature on the operational level including downstream capacity constraints.

One focus in the literature is the combination of tactical and operational decision-making. Some authors use a two-stage approach to first solve the MSS problem and then solve the surgery assignment problem. Other authors focus on an integrated approach. Makboul et al. [24] develop a robust two-stage approach with uncertainty. They use data from a mid-sized French hospital to demonstrate the tradeoff between conservatism and robustness. Aringhieri et al. [2] present a two-level metaheuristic determining the MSS and the surgery scheduling in an integrated approach using real data from a hospital in Italy. They especially focus on weekend days where elective surgeries are not performed and show that good planning in the OT can utilize the reduced number of patient admissions in the downstream unit. Testi and Tànfani [33] develop a 0–1 linear program that maximizes the utilization in the OT while considering downstream capacity and waiting list priority across specialties. The model is an integrated approach combining tactical and operational decisions. In their case study, using data from a hospital in Italy, they perform multiple “what if” scenarios by increasing the block size or number to show the influence on the objective. Li et al. [23] develop a goal programming approach to minimize the waiting time of patients, idle time in the OT, and the maximum number of required recovery beds. They demonstrate the tradeoffs between each of these goals in a case study.

Another focus in the literature is the sole focus on the tactical level. Part of the literature is concerned with the development of a mathematical model; some authors follow a combined simulation and optimization approach. The following works focus on optimization models that minimize bed variability on downstream departments. Beliën and Demeulemeester [5] build a nonlinear integer programming (IP) model to determine an MSS that minimizes the expected bed shortage in the wards. The model assigns predefined MSS blocks to rooms and days for each specialty. Adan et al. [1] develop a MIP to find an MSS that minimizes the deviations between the planned and realized resource consumption in downstream departments. They use stochastic LOS distributions based on historical data. Van Houdenhoven et al. [35] develop a MIP and apply the model to a dataset of a large hospital in the Netherlands. They show that the use of an MSS is not only beneficial to the utilization of the OT but also positively influences the leveling of workload in downstream departments, in this case, the ICU. Van Essen et al. [34] present an IP model to reduce the maximum number of occupied beds in downstream departments, and therefore the variance of bed demand. They use a local search heuristic and a simulated annealing approach to solve their model. They show that weekend days with no elective surgeries heavily influence the maximum number of occupied beds. Marques et al. [25] combine the strategic and tactical level by introducing case mix planning into the development of a new MSS while considering two downstream units. Britt et al. [8] present a hierarchical approach consisting of seven goals: minimizing idle time, variations of assignments to surgeons across weekdays, the total number of expected patients in the recovery ward, variations in the recovery ward utilization, differences from planned and realized surgeries all while meeting external waiting time targets. Our approach is to level the workload in downstream units while maintaining the current MSS to earn the support of all involved medical specialties for the implementation of the new surgery scheduling policy.

Further objectives in the literature on the tactical level are either the minimization of costs or the maximization of throughput considering the resources in the OT and the ones in downstream units. Fügener et al. [14] advance the approach by Vanberkel et al. [38] to calculate the bed demand in multiple downstream departments of the OT, i.e. multiple wards and the ICU. They present a model that minimizes the costs in downstream departments, which is solved with exact and heuristic algorithms. Fügener [15] presents an IP using stochastic patient demand on downstream units. The goal of the model is the maximization of revenue by determining the position and number of blocks in a cyclic MSS. Fügener et al. [16] further enhance their previous work by also implementing emergency cases on weekend days. They compare the current MSS of a large hospital in Germany with two generated MSS and show the influence of each MSS on the bed utilization of downstream wards.

Concerning the types of methodology that have been applied, many papers use a combined simulation and optimization approach to test the robustness of their solutions. Chow et al. [12] use a combination of Monte Carlo Simulation and a MIP model. The goal of the MIP is to minimize the bed occupancy variability by scheduling both surgeon blocks and patient types in the OT. The simulation model is used to predict the bed requirements for a specific schedule. Cappanera et al. [10] develop a MIP model to compare three scheduling policies. They use multiple performance criteria and use a discrete event simulation to test their results for robustness. Their results show that no policy is superior in all measured performance criteria. Heider et al. [19] develop a MIP model to schedule groups of elective patients in the OT to balance the expected bed occupancy in the ICU. Using a simulation model, they show that more balanced occupancy levels in the ICU are possible without changing the MSS. Rachuba et al. [27] develop a MIP combined with a simulation model to build a cyclic surgery plan with blueprints of surgical groups that result in improved OT and ICU utilization.

The current literature on the tactical level is mostly focused on generating a completely new MSS to better level workload or bed demand in downstream units. Few papers focus on surgical groups to better control the inflow of patients in downstream units (e.g. [10, 19, 27, 30]). We summarize the main features of these works in Table 1 to compare our study with the most relevant papers. The literature combining the tactical and operational level is not considered in this comparison due to the missing cyclical approach. As shown in Table 1, to the best of our knowledge, no study has provided results on the impact of surgical schedule optimization on downstream units measured before and after implementation at a hospital. Additionally, we are the first to build a model using patient quotas dependent on the downstream unit.

**Table 1:** Overview of the features of selected other studies

Authors	Downstream unit types	Optimization model	Simulation model	Heuristic	Downstream objective	# Instances	Data source	Downstream quota on tactical level	Maintain current MSS	Implementation
Beliën & Demeulemeester (2007) [5]	1 (N)	✓		✓	B	384	S			
Van Houdenhoven et al. (2008) [35]	1 (I)			✓	W	1	R			
Adan et al. (2009) [1]	2 (I, M)	✓			W	1	R			
Vanberkel et al. (2011) [38]	1 (I)	✓			W	1	R			(✓)**
Chow et al. (2011) [12]	3 (I, M, W)	✓	✓		B	1	R			(✓)**
Van Essen et al. (2014) [34]	1 (N)	✓		✓	B	100	R			
Fügener et al. (2014) [14]	2 (I, W)	✓		✓	C	3	R			
Cappanera et al. (2014) [10]	1 (W)	✓	✓		B	27	R			
Marques et al. (2019) [25]	2 (N)	✓			W	3	R			
Heider et al. (2020) [19]	1 (I)	✓	✓		B	1	R		**✓**	
Schneider et al. (2020) [30]	2 (I, W)	✓	✓		B	2	R			
Rachuba et al. (2021) [27]	1 (I)	✓	✓		-	4	S		(✓)*	
**Our Work**	**2 (I, W)**	✓	**✓**		**W**	**65**	**R**	**✓**	**✓**	**✓**

**Downstream unit types:** N = not specified, I = ICU, M = IMC, W = ward **Downstream objective:** B = bed leveling, C = costs, W = workload leveling **Data source:** S = synthetic data, R = real data *: only surgical groups within one specialty are considered. **: no data after implementation analyzed

The goal of our approach is threefold. 1) Maintaining the current MSS to prevent operational disruptions and potential disputes over surgery capacity between the involved medical specialties. 2) Introducing a quota system for downstream capacity on the tactical level while maintaining the autonomy of scheduling on an operational level at each specialty. 3) Reducing peak workloads in downstream units to reduce the workload variability.

The efficacy of ICU quotas for elective patients has been shown on an operational level by Kim and Horowitz [22]. They show that daily elective surgery quotas reduce the number of canceled surgeries, therefore improving the utilization of the OT and the ICU. In this paper, we extend the idea of Kim and Horowitz and integrate the quotas for ICU patients in the MSS on a tactical level. We introduce an extension to the MSS by using distinct block types for individual downstream units to reduce the peak workload in those units while maintaining the scheduling autonomy of each specialty. The introduction of individual block types can be seen as an alternate form of surgical groups, where groups are built depending on the specialty and the medically required downstream department of elective patients. Additionally, the model presented in this paper was used to support the implementation of an ICU quota system in the OT. Actual implementation of operations management (OM) methods in practice is limited to this date in the healthcare sector, as recently noted by Keskinocak & Savva [21], especially in the OT. Few examples of at least partial implementation of surgery scheduling on the tactical level are Blake and Donald [7], Ozen et al. [26], and Visintin et al. [39] with an isolated view of the OT without including recovery units, as well as Chow et al. [12] and Vanberkel et al. [38] which focus on creating a new MSS to level workload in downstream units.

## Methodology

In this section, we define the functionality of our approach, followed by the problem description and our proposed mathematical model. Then, we describe our solution approach including the simulation model used for the evaluation of our results.

### Problem Setting and Problem Description

**Fig. 1:**
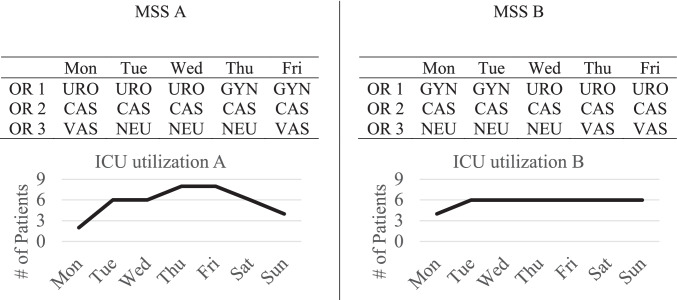
Two MSS examples with resulting ICU bed utilization

An MSS is a cyclic plan usually repeated every one or two weeks. In the MSS, OT capacity is assigned to clinical specialties in blocks. In most hospitals, one block has the size of one day. Two illustrative MSSs with three ORs can be seen in Fig. 1. In MSS A, for example, Urology is assigned to OR 1 on Mondays, Tuesdays, and Wednesdays for the whole day. On Thursdays and Fridays, Gynecology performs surgeries in OR 1. The research idea of MSS scheduling with consideration of downstream utilization is demonstrated at the bottom of Fig. 1, where we graph the resulting expected ICU bed demand of MSS A (left part) and MSS B (right part). The goal is to reschedule the blocks in the OT in a way that the resulting average bed demand in the ICU or the bed wards is as most balanced as possible. This is possible because the length of stay (LOS) in the ICU and the general wards varies for each specialty. Current literature often assumes a fixed share of ICU and ward patients in every block of a specialty based on historical data. In this example, MSS B shows better leveling than MSS A.

According to Van Oostrum et al. [36], the MSS combines the advantages of a centralized and a decentralized planning approach when scheduling surgeries in the OT. The most important benefits when using an MSS are that surgeons keep their autonomy, e.g., when selecting and sequencing patients in their respective surgery blocks. This allows fast decision-making when surgeries take longer or shorter than excepted and when urgent or emergency cases arrive. When an MSS is set up for the first time, a substantial amount of data is needed for capacity planning to assign OT blocks to specialties. This procedure needs to be repeated over time to reevaluate the capacity planning for all specialties in the OT. On a tactical level, an MSS reduces the communication and coordination efforts, since the system complexity is reduced compared to a fully centralized approach. Most of the operational control is handed to the surgeons. Surgeons are employed by the specialties so that patients can be moved freely between surgeons within each specialty – a common setup in most European hospitals [13]. The cyclic nature of an MSS allows easy integration of multiple planning processes as well. The fixed setup of assigning specialties to rooms on given days leads to robustness against cheating and high OT utilization levels. An MSS offers reasonable predictability of patient flows regarding the number of patients operated on each day in each specialty, but not on the type of patient within a specialty. The type of patient, however, is extremely important for predicting the patient flow within the hospital. Severe cases are transferred to the ICU after surgery, less severe cases to the general bed wards. The creation of a new MSS can lead to reduced expected variability of bed demand or workload in downstream units on a tactical level. On an operational level, however, additional managerial coordination is necessary to control the patient flow.

**Fig. 2:**
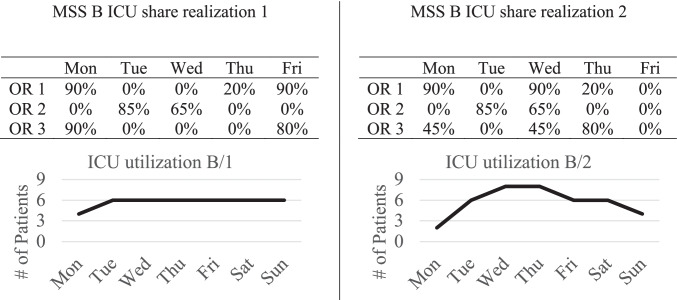
Two possible realizations of the ICU share in the OT and the resulting ICU utilization with MSS B

Fig. 2 illustrates this problem. In this example, MSS B (as shown in Fig. 1 on the right) is used. The percentages in the tables show the share of ICU patients operated in each block. While the share of ICU patients in realization 1 (left part) leads to the exact ICU bed demand forecasted in Fig. 1, the ICU bed demand for realization 2 is drastically different from the forecast. The problem is that with no additional rules or quotas, specialties have full autonomy and can schedule severe cases with a high probability of being transferred to the ICU in every block that is assigned to the specialty. Independent planning of specialties can lead to higher than average demand on some days and to lower than average demand on other days.

Current research, however, assumes that the share of ICU patients is equal in each block of a given specialty, namely the average or a distribution based on historical data. We relax this assumption. For general bed wards, this problem is not as serious as for the ICU since specialties have a good overview of available beds and the status of recovery of the patients in their respective wards. In the ICU, however, a shared unit between all specialties, the decentralized planning of each specialty can result in high bed utilization variability, causing canceled surgeries or early discharges when no ICU beds are available. Additionally, a vast operational coordination effort is necessary to manage the decentral planning of each specialty for ICU patients in the OT. Nevertheless, the ICU share in each block influences the regular bed wards, since the remaining share of patients represents those transferred to the regular wards, which can lead to a high variability as well.

The general MSS approach allows specialties to schedule ICU and ward patients in every block assigned to them which may lead to overbooking of available ICU beds on some days. The absence of these controlling mechanisms on the tactical level requires additional work on the operational level due to the daily rescheduling of surgeries to not exceed the capacity in the recovery wards. Work for limiting and rescheduling the number of ICU patients is often not supported by a computer system which leads to a multitude of phone calls and handwritten lists. Without these manual mechanisms, an even higher variability of ICU and ward admissions is likely, which would result in unnecessarily large fluctuations of workload and bed demand. We address this problem by introducing multiple block types for individual downstream resources to better control the patient flow on a tactical level, i.e., ward blocks and ICU blocks. In a ward block, only surgeries of ward patients should be scheduled. In an ICU block, the available time may be freely assigned to ICU patients and ward patients. The likelihood of an ICU transfer can be retrieved from historical data and is usually determined by an anesthesiologist beforehand. Recent studies show that machine learning algorithms can successfully support physicians in forecasting patients’ pathways post-surgery [29]. The block type for each specialty, day, and room is determined with the mathematical model described in Section 3.2.

**Fig. 3:**
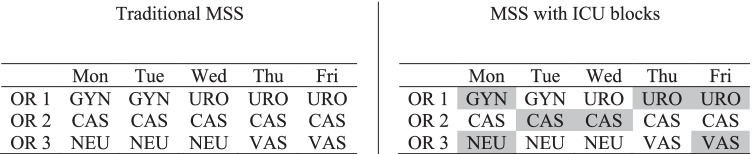
Example of a traditional MSS next to an MSS with ICU blocks, shaded grey

In Fig. 3, we illustrate the difference between a traditional MSS and our approach using MSS B from Fig. 1 (right-hand side). On top of the assignment of ORs to specialties on each day, there is also an indication of whether or not ICU patients can be scheduled within each block. ICU blocks are shaded in grey. Ward patients can be operated on in both ICU and ward blocks.

**Fig. 4:**
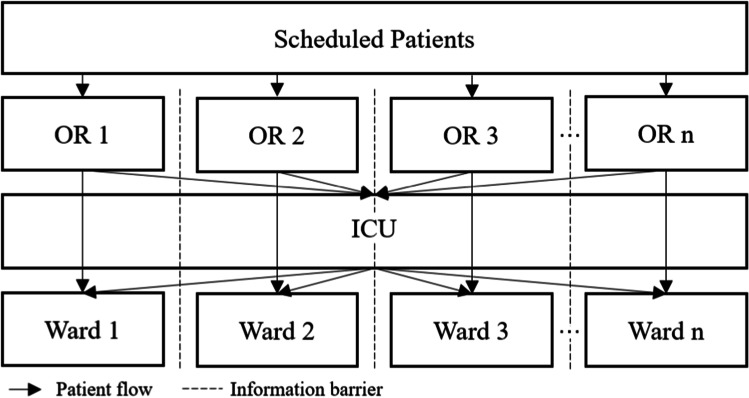
Simplified patient flow of the mathematical model with ICU as the bottleneck

As previously stated, the absence of control over the patient flow on a tactical level is especially critical in the ICU, a shared downstream resource of all clinical specialties. We illustrate this problem in Fig. 4, showing a simplified version of possible patient paths where each specialty has only one OR and one ward station. ICU patients in the OT are first transferred to the ICU and then later to the regular bed ward of each specialty. Regular patients are directly transferred to the regular bed ward of each specialty. According to our data, 88% of all patients with at least one surgery took one of the two modeled paths. Hence, the chosen simplification of patient flow strikes a balance between realism and model complexity reduction.

The goal of the MIP is to find an MSS with distinct block types for each downstream unit, which minimizes the maximum workload for physicians and nurses in the ICU and the general bed wards. Additionally, the number of ICU blocks is minimized to implicitly introduce daily ICU quotas for each specialty. The workload for physicians and nurses as well as the number of ICU blocks are weighted in the objective function, allowing the decision-maker to find an appropriate balance between flexibility and peak workload reduction. We discuss the influence of the weights on the solution of the model in a sensitivity analysis in Appendix C (online supplements). For modeling purposes, an ICU share is introduced in each block, which can only be larger than zero if a block is an ICU block. The average share over all blocks has to meet a total ICU share for each specialty, which may, e.g., be based on historical demand. The share of ICU patients in each block represents the mathematically optimal solution to minimize the maximum workload in downstream units as best as possible. The workload in downstream units is calculated separately for nurses and physicians. Nurses and physicians work in three shifts representing the early, late, and night shift. The nurse workload in downstream departments is determined by a patient-to-nurse ratio for each shift, similar to regulatory requirements in other countries [31]. Since a patient to physician ratio is not suggested in the literature or by regulatory requirements, we assign a workload for admissions, daily routine work, and discharges for every shift in the ICU and the ward stations of each specialty. This total workload in every shift is then divided by the working hours to determine the full-time equivalent (FTE) of physicians required to be present in each shift. The workload associated with admissions and discharges falls into the early shift (e.g., 7:30 am to 4:30 pm) of each day. The discharge probabilities and probability distribution of patient length of stay in the ICU and the bed wards are calculated during preprocessing and are rounded to whole days. The process is described in detail in Section 3.2 and Appendix B (online supplements). Other specific parameters for each specialty can either be derived from historical data or should be determined on a strategic level. These parameters include: the average number of patients in each block; the number of blocks in the planning horizon; the maximum number of daily blocks; the patient-to-nurse ratio in every shift for the regular bed wards; the physician workload for admissions and discharges in the regular bed wards; additionally, an existing MSS can be used as input parameter if it should be maintained. Other parameters are the patient-to-nurse ratio in the ICU as well as the physician workload for admissions, daily routine, and discharges in the ICU.

### Mathematical Model and Evaluation Procedure

**Fig. 5:**
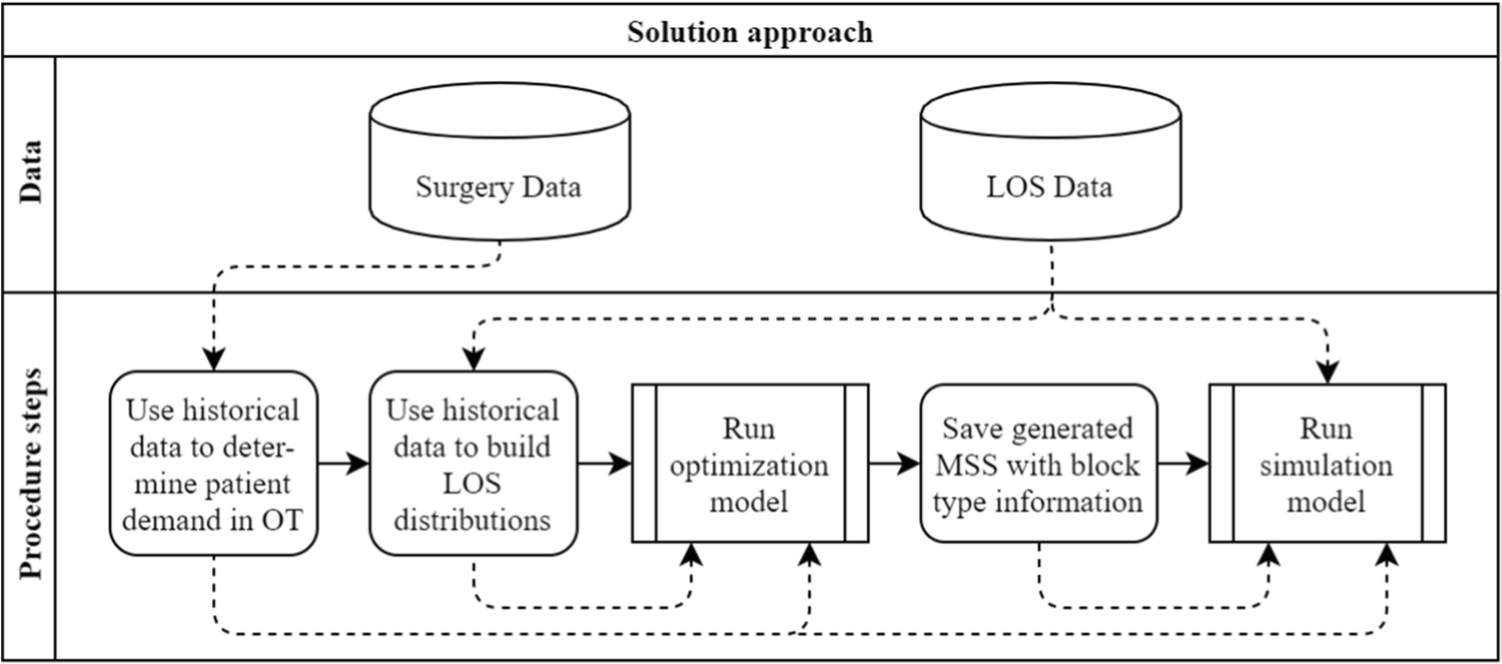
Overview of solution and evaluation approach

In the following section, we explain the mathematical model, the solution procedure, and our evaluation approach. First, we describe the process to derive the LOS distributions for the optimization model. Second, we present our mathematical model to generate an MSS with distinct block types. Third, we illustrate our simulation model, which is used to evaluate the operational workload that derives from the tactical MSS calculated in the optimization model. Our overall solution approach is shown in Fig. 5. All solid arrows represent the connection to the next step in the solution approach, all dashed arrows represent data flows.

In the first preprocessing step, patient demand in the OT is determined. The average number of patients in a block ${P}_{c}^{\#}$ and the ICU share ${P}_{c}^{\mathrm{Share}}$ for each specialty $c\in C$ are calculated from historical data. The number of blocks per specialty ${B}_{c}^{\mathrm{Total}}$ and the maximum number of daily blocks per specialty ${B}_{c}^{\mathrm{Max}}$ are determined by the preexisting MSS.

Additional historical data required in preprocessing are the probabilities that a patient of specialty $c$ stays $q\in Q$ days after surgery in the ICU ${(d}_{c,q}^{\mathrm{ICU}})$ as well as the probabilities that a patient of specialty $c$ stays $q$ days in the ward after surgery or a transfer from the ICU ${(d}_{c,q,i}^{\mathrm{Ward}})$ with $q=0$ for the day of surgery or the transfer from the ICU. Since the decision of admission or discharge is made once per day, historical LOS values are rounded to whole days to build the LOS distributions. From these probabilities, the LOS distributions for the ICU ${(l}_{c,q}^{\mathrm{ICU}}$) and the general bed wards ${(l}_{c,q,i}^{\mathrm{Ward}})$ can be calculated. The parameters ${l}_{c,q}^{\mathrm{ICU}}$ and ${l}_{c,q,i}^{\mathrm{Ward}}$ are the cumulative distribution functions of ${d}_{c,q}^{\mathrm{ICU}}$ and ${d}_{c,q,i}^{\mathrm{Ward}}$.They represent the probability that a patient stays at least $q$ days in the ICU after surgery or $q$ days in a bed ward after surgery or a transfer from the ICU. Finally, all probabilities are convolved into the planning horizon to allow for the cyclic approach of our proposed MIP. The calculations are shown in Appendix B (online supplements).

The presented model is focused on the tactical level, thus aiming to find an optimal MSS that will be maintained over months or even years. Therefore, the number of patients in each block and the ICU share in a block are both assumed to be continuous. This is also true for the LOS and discharge probabilities, which determine the share of patients who are still in the ICU or the regular wards after a certain number of days. The model uses a cyclic approach. Hence, patients with a LOS that exceeds the planning horizon will be added to the beginning of the planning horizon. The goal is to minimize the weighted maximum workload measured in FTEs in each shift – early, late, and night – in the planning horizon to reduce the workload variability as well as the weighted number of ICU blocks for each specialty to implicitly limit the number of ICU patients on an operational level.

**Table:** 

Index $\in$ set	Description
$c\in C$	Specialties
$r\in R$	OT-Rooms
$t\in T$	Days in the planning horizon
$t\in O$	Days without elective surgery,$O\subseteq T$
$i\in I$	Block or Patient type (Ward block = 0 or ICU block = 1)
$s\in S$	Shifts in a day (early, late, night)
Parameters	
${L}_{c,t}^{\mathrm{ICU}}$	Probability that a patient from specialty $c$ stays at least $t$ days in the ICU
${E}_{c,t}^{\mathrm{ICU}}$	Probability that a patient from specialty $c$ stays exactly $t$ days in the ICU
${L}_{c,t,i}^{\mathrm{Ward}}$	Probability that a patient from specialty $c$ stays at least $t$ days in the ward with prior transfer from the ICU $(i=1)$ or OT $(i=0)$
${E}_{c,t,i}^{\mathrm{Ward}}$	Probability that a patient from specialty $c$ stays exactly $t$ days in the ward with prior transfer from the ICU $(i=1)$ or OT $(i=0)$
${B}_{c}^{\mathrm{Max}}$	Maximum number of daily MSS blocks for specialty $c$
${B}_{c}^{\mathrm{Total}}$	Required number of MSS blocks in the planning horizon for specialty $c$
${P}_{c}^{\#}$	Average number of patients in each block for specialty $c$
${P}_{c}^{\mathrm{Share}}$	Target ICU share for specialty *c* over the planning horizon
${N}_{s}^{\mathrm{ICU}}$	Patient-to-nurse ratio in the ICU in shift $s$
${N}_{c,s}^{\mathrm{Ward}}$	Patient-to-nurse ratio in ward $c$ in shift $s$
${A}_{s}^{\mathrm{ICU}}$	Physician time associated with an ICU admission in shift $s$
${R}_{t,s}^{\mathrm{ICU}}$	Physician time associated with ICU daily routine for each patient on day $t$ in shift $s$
${D}_{s}^{\mathrm{ICU}}$	Physician time associated with a discharge from the ICU in shift $s$
${A}_{c,s}^{\mathrm{Ward}}$	Physician time associated with a patient admission in a ward with specialty $c$ in shift $s$
${R}_{c,t,s}^{\mathrm{Ward}}$	Physician time associated with daily ward routine for each patient in time slot $t$ for specialty $c$ in shift $s$
${D}_{c,s}^{\mathrm{Ward}}$	Physician time associated with a discharge from a ward with specialty $c$ in shift $s$
${H}_{t,s}$	Shift length for ICU and ward stations on day $t$ in shift $s$
${\overline{M} }_{c,r,t}$	Preexisting MSS assignments (if necessary); 1 if specialty $c$ has room $r$ on day $t$, 0 otherwise
$\alpha$	Penalty weight for the maximum nurse workload in the ICU and wards
$\beta$	Penalty weight for the maximum physician workload in the ICU and wards
$\gamma$	Penalty weight for the number of ICU blocks
Decision variables	
${m}_{c,r,t,i}$	1 if specialty $c$ has room $r$ on day $t$ with block type $i$, 0 otherwise
${b}_{c,r,t}^{\mathrm{Share}}$	ICU share in a block of specialty $c$ in room $r$ on day $t$
${n}_{t,s}^{\mathrm{ICU}}$	FTE of ICU nurses required on day $t$ in shift s
${n}_{s}^{\mathrm{ICUMax}}$	Maximum FTE of ICU nurses in shift $s$ within the planning horizon
${p}_{t,s}^{\mathrm{ICU}}$	FTE of ICU physicians required on day $t$ in shift $s$
${p}_{s}^{\mathrm{ICUMax}}$	Maximum FTE of ICU physicians in shift $s$ within the planning horizon
${n}_{c,t,s}^{\mathrm{Ward}}$	FTE of ward nurses required for specialty $c$ on day $t$ in shift $s$
${n}_{c,s}^{\mathrm{WardMax}}$	Maximum FTE of ward nurses for specialty $c$ in shift $s$ within the planning horizon
${p}_{c,t,s}^{\mathrm{Ward}}$	FTE of ward physicians for specialty $c$ on day $t$ in shift $s$
${p}_{c,s}^{\mathrm{WardMax}}$	Maximum FTE of ward physicians for specialty $c$ in shift $s$ within the planning horizon
${y}_{c,t,i}$	Auxiliary variable for the ward admissions of patient type *i* specialty $c$ on day $t$
${z}_{c,r,t}$	Auxiliary variable for linearization

1 $$\begin{array}{c}\mathrm{min}\alpha \cdot \sum\limits_{s\in S}\left({n}_{s}^{\mathrm{ICUMax}}+\sum\limits_{c\in C}\left({n}_{c,s}^{\mathrm{WardMax}}\right)\right)+\beta \cdot \sum\limits_{s\in S}\left({p}_{s}^{\mathrm{ICUMax}}+\sum\limits_{c\in C}\left({p}_{c,s}^{\mathrm{WardMax}}\right)\right)+\\ + \gamma \cdot \sum\limits_{c\in C}\sum\limits_{r\in R}\sum\limits_{t\in T}{m}_{c,r,t,1}\end{array}$$

s.t.

2 $$\sum_{r\in R}\sum_{t\in T}\sum_{i\in I}{m}_{c,r,t,i}={B}_{c}^{\mathrm{Total}} \forall c\in C$$

3 $$\sum_{r\in R}\sum_{i\in I}{m}_{c,r,t,i}\le {B}_{c}^{\mathrm{Max}} \forall c\in C, t\in T$$

4 $${m}_{c,r,t,i}\le 0 \forall c\in C, r\in R,t\in O, i\in I$$

5 $$\sum_{c\in C}\sum_{i\in I}{m}_{c,r,t,i}\le 1 \forall r\in R,t\in T$$

6 $${b}_{c,r,t}^{\mathrm{Share}}\le {m}_{c,r,t,1} \forall c\in C, r\in R,t\in T$$

7 $$\frac{\sum_{r\in R}\sum_{t\in T}{b}_{c,r,t}^{\mathrm{Share}}}{{B}_{c}^{\mathrm{Total}}}={P}_{c}^{\mathrm{Share}} \forall c\in C$$

8 $$\sum_{c\in C}\sum_{r\in R}\sum_{k\in T}{L}_{c,k}^{\mathrm{ICU}}\cdot {P}_{c}^{\#}\cdot {b}_{c,r,t-k}^{\mathrm{Share}}\le {N}_{s}^{\mathrm{ICU}}\cdot {n}_{t,s}^{\mathrm{ICU}} \forall t\in T, s\in S$$

9 $${n}_{t,s}^{\mathrm{ICU}}\le {n}_{s}^{\mathrm{ICUMax}}\forall t\in T, s\in S$$

10 $$\begin{array}{c}\sum\limits_{c\in C}\sum\limits_{r\in R}{A}_{s}^{\mathrm{ICU}}\cdot {P}_{c}^{\#}\cdot {b}_{c,r,t}^{\mathrm{Share}}+\sum\limits_{c\in C}\sum\limits_{r\in R}\sum\limits_{k\in T}{R}_{t,s}^{\mathrm{ICU}}\cdot {P}_{c}^{\#}\cdot {L}_{c,k}^{\mathrm{ICU}}\cdot {b}_{c,r,t-k}^{\mathrm{Share}}+\\ +\sum\limits_{c\in C}\sum_{r\in R}\sum\limits_{k\in T}{D}_{s}^{\mathrm{ICU}}\cdot {P}_{c}^{\#}\cdot {E}_{c,t}^{\mathrm{ICU}}\cdot {b}_{c,r,t-k}^{\mathrm{Share}}\le {H}_{t,s}\cdot {p}_{t,s}^{\mathrm{ICU}} \forall t\in T, s\in S\end{array}$$

11 $${p}_{t,s}^{\mathrm{ICU}}\le {p}_{s}^{\mathrm{ICUMax}} \forall t\in T, s\in S$$

12 $$\sum_{r\in R}\sum_{k\in T}{P}_{c}^{\#}\cdot {E}_{c,t}^{\mathrm{ICU}}\cdot {b}_{c,r,t-k}^{\mathrm{Share}}={y}_{c,t,1} \forall c\in C,t\in T$$

13 $$\sum_{r\in R}{P}_{c}^{\#}\cdot \left({m}_{c,r,t,0}+\left(1-{b}_{c,r,t}^{\mathrm{Share}}\right)\cdot {m}_{c,r,t,1}\right)={y}_{c,t,0} \forall c\in C,t\in T$$

14 $$\sum_{i\in I}\sum_{k\in T}{L}_{c,k,i}^{\mathrm{Ward}}\cdot {y}_{c,t-k,i}\le {N}_{c,s}^{\mathrm{Ward}}\cdot {n}_{c,t,s}^{\mathrm{Ward}} \forall c\in C, t\in T, s\in S$$

15 $${n}_{c,t,s}^{\mathrm{Ward}}\le {n}_{c,s}^{\mathrm{WardMax}} \forall c\in C, t\in T, s\in S$$

16 $$+\begin{array}{c}\sum\limits_{i\in I}{A}_{c,s}^{\mathrm{Ward}}\cdot {y}_{c,t,i}+\sum\limits_{i\in I}\sum\limits_{k\in T}{R}_{c,t,s}^{\mathrm{Ward}}\cdot {L}_{c,k,i}^{\mathrm{Ward}}\cdot {y}_{c,t-k,i}+\\ +\sum\limits_{i\in I}\sum\limits_{k\in T}{D}_{c,s}^{\mathrm{Ward}}\cdot {E}_{c,t,i}^{\mathrm{Ward}}\cdot {y}_{c,t-k,i}\le {H}_{t,s}\cdot {p}_{c,t,s}^{\mathrm{Ward}} \forall c\in C, t\in T, s\in S\end{array}$$

17 $${p}_{c,t,s}^{\mathrm{Ward}}\le {p}_{c,s}^{\mathrm{WardMax}} \forall c\in C, t\in T, s\in S$$

18 $${m}_{c,r,t,i} \{\mathrm{0,1}\}$$

19 $${b}_{c,r,t}^{\mathrm{Share}},{n}_{t,s}^{\mathrm{ICU}},{n}_{s}^{\mathrm{ICUMax}},{p}_{t,s}^{\mathrm{ICU}},{p}_{s}^{\mathrm{ICUMax}},{n}_{c,t,s}^{\mathrm{Ward}},{n}_{c,s}^{\mathrm{WardMax}},{p}_{c,t,s}^{\mathrm{Ward}},{p}_{c,s}^{\mathrm{WardMax}},{y}_{c,t,i}\ge 0$$

The objective function (1) minimizes the weighted maximum number of nurses in the ICU and bed wards for each specialty in every shift, the weighted maximum number of physicians in the ICU and bed wards of each specialty in every shift, and the weighted number of ICU blocks. The number of nurses and physicians as well as the maximum number of nurses and physicians are measured as FTE. The constraints consist of three major groups. The first group, Constraints (2) to (7), represents all constraints required for the OT. The second group, Constraints (8) to (11), deals with patient flow and workload in the ICU. Finally, Constraints (12) to (17) handle the patient flow through the ward stations as well as the workload.

Constraints (2) and (3) assure that the number of weekly blocks for each specialty is maintained and the number of daily blocks for each specialty is not exceeded. Blocks should not be scheduled on weekend days (4). Constraints (5) ensure that a room can be assigned to no more than one specialty and must be used either as an ICU block or a ward block. Constraints (6) assure that a block can only have a positive ICU share if the block is an ICU block. Constraints (7) ensure that the total share of ICU patients has to meet the target share for each specialty.

Constraints (8) calculate the nurse workload in the ICU for every shift. Nurse workload is determined by a patient-to-nurse ratio in the ICU. The subscript $t-k$ in these and the following constraints is treated as the modulo of the surgery cycle length $\left(t-k\right) mod \left|T\right|$ to represent the cyclic approach. Constraints (9) compute the maximum nurse workload in every shift over the planning horizon. Constraints (10) are similar to Constraints (8), only that the workload of physicians in every shift is calculated. Instead of a physician-to-patient ratio, the amount of work for admissions, daily routine, and discharges is computed and divided by the hours worked by one physician. Similar to Constraints (9), the maximum physician workload in every shift over the planning horizon is calculated in Constraints (11).

Constraints (12) and (13) calculate the inflow of patients to the ward stations from the ICU and the OT, respectively. Constraints (14) to (17) are similar to Constraints (8) to (11), only that in this case the workload, as well as the maximum workload for nurses and physicians, are calculated for the ward units instead of the ICU. Constraints (18) and (19) define the domain of all decision variables.

Due to the nonlinear nature of Constraints (13), we replace these constraints with a linear reformulation shown in (20)—(24).

20 $${z}_{c,r,t}\le {m}_{c,r,t,1} \forall c\in C, r\in R,t\in T$$

21 $${z}_{c,r,t}\le 1-{b}_{c,r,t}^{\mathrm{Share}} \forall c\in C, r\in R,t\in T$$

22 $${z}_{c,r,t}\ge \left(1-{b}_{c,r,t}^{\mathrm{Share}}\right)-\left(1-{m}_{c,r,t,1}\right) \forall c\in C, r\in R,t\in T$$

23 $$\sum_{r\in R}{P}_{c}^{\#}\cdot \left({m}_{c,r,t,0}+{z}_{c,r,t}\right)={y}_{c,t,0} \forall c\in C,t\in T$$

24 $${z}_{c,r,t}\ge 0$$

In settings where the room assignments must remain unchanged from an existing MSS, Constraints (25) are added to the model.

25 $$\sum_{i\in \mathrm{I}}{m}_{c,r,t,i}={\overline{M} }_{c,r,t} \forall c\in C,r\in R, t\in T$$

If a new MSS should be calculated, Constraints (26) are added to the model. In this case, all blocks are ICU blocks since ICU patients can be scheduled in every block.

26 $${b}_{c,r,t}^{\mathrm{Share}}={P}_{c}^{\mathrm{Share}}\cdot {m}_{c,r,t,1} \forall c\in C,r\in R, t\in T$$

After an optimal MSS is obtained by solving the mathematical model, we use a simulation model to evaluate the resulting workload for nurses and physicians in the downstream departments on an operational level, drawing from historical patient data. In the simulation model, we use integer values for the number of patients in a block as well as for the LOS in downstream units. The share in each block can lead to results that cannot be realized in practice, e.g., 1% ICU patients in a block with 5 total patients. Therefore, in the simulation model, we only consider the block type of each block and neglect the shares. Consequently, the ICU capacity in the OT is only implicitly limited, not explicitly.

**Fig. 6:**
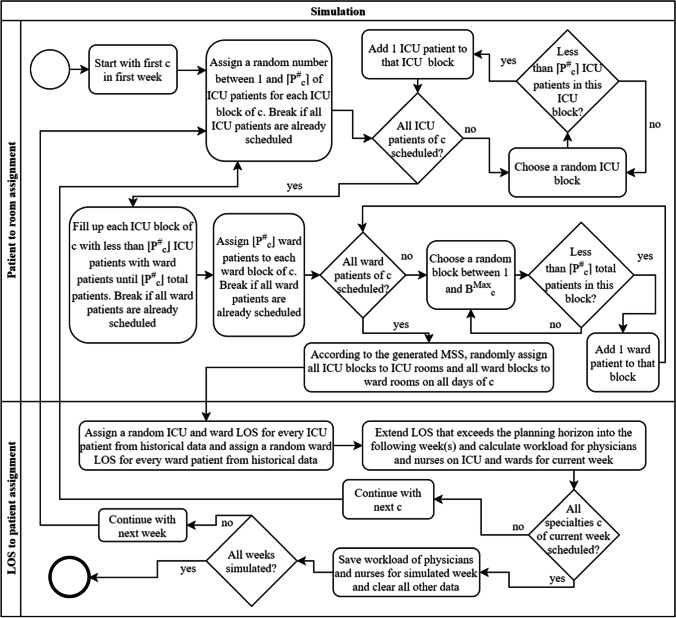
Flowchart of the simulation procedure for MSS with distinct block types

A flowchart of the simulation procedure is shown in Fig. 6. The model simulates 50,000 consecutive weeks so that the resulting confidence intervals around the reported averages are sufficiently small. The maximum workload is evaluated for each week separately. The number of patients per block is random but limited to a maximum level, depending on the specialty. The number of ICU patients in a block is random as well. ICU patients can only be scheduled in ICU blocks and ward patients in both blocks. The simulation is built for our approach using an MSS with distinct block types. If a regular MSS needs to be simulated, all blocks are labeled as ICU blocks, since ward patients can be scheduled in every block, as already mentioned in the description of Constraints (26).

Each of the 50,000 simulation runs is divided into two parts. In the first part, all ICU and ward blocks of each specialty are randomly filled with ICU and ward patients according to the block type. The blocks are not yet assigned to a day and room. The block-to-day-and-room assignment follows in the second step in random order according to the generated MSS from the mathematical model. Both parts are repeated until all specialties in a certain week have been simulated. The steps in a single simulation run can be described as follows: For each specialty, the first step is the random assignment of ICU patients to ICU blocks. When all ICU patients are scheduled into ICU blocks, potential remaining space is filled with ward patients. Next, all remaining ward patients are scheduled into ward blocks. All ICU and ward blocks are then randomly assigned to a room and day according to the MSS with block types determined by the mathematical model. This is done to prevent potential trends that could occur, e.g., having more ICU patients in blocks at the beginning of the planning horizon compared to the end of the planning horizon. Next, the LOS for each ICU and ward patient, as well as the ward LOS for ICU patients after being discharged from the ICU are randomly picked from historical data. This is repeated for all specialties. In total, 50,000 weeks are simulated and therefore each run, as described before, is repeated 50,000 times. Different from the formulation in the mathematical model, the LOS of patients who stay longer than the planning horizon is extended into the following week or weeks. The workload is calculated according to the mathematical model, however not all weeks of the 50,000 are included in the results since a warmup phase is needed with the length of the maximum LOS a patient could stay in the hospital i.e., 33 weeks.

## Numerical Study

In the following section, we first present a case study with data from UKA. We present the results of the mathematical model, followed by the results of the simulation model to show the influence of the tactical plans on the operational level in various analyses. The influence of weights used in the objective function is further discussed in Appendix C (online supplements). A benchmark, comparing our approach to individual surgery scheduling on the operational level, where we show that our approach can realize up to 79.85% of the maximum workload reduction in the ICU, is shown in Appendix B (online supplements). Lastly, to generalize our findings, we create 64 instances by varying the total number of ORs, the number of specialties, the number of total ICU and ward patients, as well as the number of ORs and ICU and ward patients per specialty. We use our described evaluation procedure on all 64 instances and present the results.

We analyze four different policies: the current MSS of UKA with no distinct block types (in the following: CMSS), a new MSS and no distinct block types (NMSS), the current MSS of UKA using our approach with distinct block types (CMSSB), and a new MSS with distinct block types (NMSSB). We use two different types of distinct blocks, one for the ICU and one for the general ward. For all different policies, we first calculate an MSS in the optimization model – except for CMSS – and then use our simulation model to evaluate the tactical MSS on an operational level.

We use seven years of data from UKA, one of the largest hospitals in Germany, to generate an MSS for different policies with the generic model presented in Section 3.2. The data is prepared in preprocessing as described in Section 3.2. The maximum number of daily MSS blocks for each specialty, the required number of MSS blocks for each specialty, the target ICU share for each specialty, and the average number of patients in each block for each specialty are all based on historical data. The day is divided into three eight-hour shifts. The penalty weights for the maximum nurse and physician workload are set according to the average costs of a nurse and physician: $\alpha =2$ and $\beta =3$. The weight for the number of ICU blocks $\gamma$ is set to 0.18, which we found to achieve the maximum peak workload reduction on the operational level in a sensitivity analysis which we discuss in detail in Appendix C (online supplements). The patient-to-nurse ratios for all shifts and specialties are based on the ratios recently introduced by the German government [9]. The physician workload associated with admissions, daily routine, and discharges is set according to the results of time studies performed by the hospital. In total, there are 16 rooms in the central OT with eight specialties in the current setup. Surgeons are not independent but rather are employed by a specialty, allowing for a relatively free assignment of patients to surgeons within a specialty – a feature commonly used in German hospitals. The considered planning horizon is one week.

### Results

The mathematical model is implemented in IBM ILOG CPLEX 12.8. All policies are solved to optimality within a few seconds. The solutions for each of the four policies (CMSS, CMMSB, NMSS, NMSSB) are shown on an aggregated level in Table 2. The table is divided into four quadrants, two at the top, and two at the bottom – one quadrant for every policy. The quadrants at the top display the number of ICU and ward blocks for the policies with the existing MSS. The first quadrant shows CMSS and the second one represents CMSSB. When a traditional MSS is used or generated, all blocks are ICU blocks, as already described in Section 3.2. The quadrants in the bottom show the number of ICU and ward blocks for the policies with a new MSS. The first quadrant displays NMSS, the second one NMSSB. In each quadrant are the number of ICU and ward blocks for every specialty every day, followed by the total. For example, for a new MSS with distinct block types, which is represented by the second quadrant at the bottom, there are two ICU blocks and one ward block on a Thursday for specialty 4.

**Table 2:** Overview of the number of ICU and ward blocks for each specialty on each day

ICU blocks/ ward blocks c		Traditional MSS						MSS with distinct block types					
		Mo	Tu	We	Th	Fr	**Sum**	Mo	Tu	We	Th	Fr	**Sum**
		CMSS						CMSSB					
Existing MSS	1	4/0	3/0	3/0	4/0	4/0	**18/0**	0/4	0/3	1/2	1/3	1/3	**3/15**
	2	1/0	2/0	2/0	1/0	1/0	**7/0**	0/1	0/2	1/1	0/1	0/1	**1/6**
	3	1/0	2/0	2/0	2/0	1/0	**8/0**	0/1	0/2	0/2	0/2	0/1	**0/8**
	4	3/0	3/0	3/0	3/0	3/0	**15/0**	3/0	1/2	1/2	2/1	2/1	**9/6**
	5	1/0	0/0	1/0	0/0	1/0	**3/0**	0/1	0/0	0/1	0/0	0/1	**0/3**
	6	2/0	2/0	1/0	2/0	2/0	**9/0**	0/2	2/0	0/1	0/2	0/2	**2/7**
	7	3/0	3/0	3/0	3/0	3/0	**15/0**	1/2	0/3	0/3	0/3	0/3	**1/14**
	8	1/0	1/0	1/0	1/0	1/0	**5/0**	0/1	0/1	0/1	0/1	0/1	**0/5**
	**Sum**	**16/0**	**16/0**	**16/0**	**16/0**	**16/0**	**80/0**	**4/12**	**3/13**	**3/13**	**3/13**	**3/13**	**16/64**
		NMSS						NMSSB					
New MSS	1	4/0	4/0	4/0	3/0	3/0	**18/0**	0/3	1/3	1/3	1/3	0/3	**3/15**
	2	1/0	2/0	1/0	2/0	1/0	**7/0**	0/2	0/1	1/1	0/1	0/1	**1/6**
	3	2/0	1/0	1/0	2/0	2/0	**8/0**	0/2	0/2	0/0	0/2	0/2	**0/8**
	4	3/0	3/0	3/0	3/0	3/0	**15/0**	3/0	1/2	1/2	2/1	2/1	**9/6**
	5	1/0	0/0	1/0	0/0	1/0	**3/0**	0/1	0/0	0/1	0/0	0/1	**0/3**
	6	1/0	2/0	2/0	2/0	2/0	**9/0**	1/0	1/1	0/2	0/2	0/2	**2/7**
	7	3/0	3/0	3/0	3/0	3/0	**15/0**	0/3	0/3	0/3	0/3	1/2	**1/14**
	8	1/0	1/0	1/0	1/0	1/0	**5/0**	0/1	0/1	0/1	0/1	0/1	**0/5**
	**Sum**	**16/0**	**16/0**	**16/0**	**16/0**	**16/0**	**80/0**	**4/12**	**3/13**	**3/13**	**3/13**	**3/13**	**16/64**

In all policies, all 80 total blocks are scheduled, covering all 16 rooms on five days of the week. For both the existing MSS with distinct block types and the new MSS with distinct block types, there are 16 ICU blocks in total. The reduction of blocks in which ICU patients could be scheduled from 80 to 16 leads to better control of the patient flow from the OT to the recovery units, especially in the ICU which we show later. Single days are different for both policies, e.g. Specialty 1, whereas in CMSSB the ICU blocks are scheduled from Wednesday to Friday and in NMSSB from Tuesday to Thursday. This is similar to other specialties and ward blocks.

Overall, there is little change for both new MSS, with or without distinct block types, compared to the CMSS. The main reason for this small change is the maximum number of daily blocks per specialty. Even though the workload within the OT is not minimized in the objective function, it is still considered through Constraints (1.3). Usually, hospitals try to distribute the blocks of each specialty as best as possible throughout the planning horizon. If the blocks of some specialties would not be more or less evenly distributed, more physicians, anesthetists, and nurses would be required on some days to handle the demand in the OT and would be idle on other days. In multiple what-if scenarios, [34] and [19] show how the violation of various necessary constraints limiting the resource availability in the OT can benefit the bed or workload leveling on downstream units. Relaxing these constraints would therefore have the potential of better workload leveling in downstream units but would also result in imbalanced OT workload levels.

**Fig. 7:**
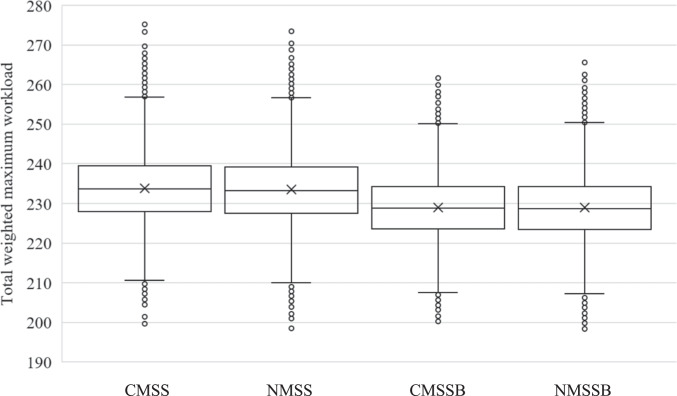
Comparison of the total weighted maximum workload for each policy

In the next step, we use our simulation model to evaluate the workload on an operational level that results from each MSS of the four policies. The workflow of the simulation is described in detail in Section 3.2. The results of the simulation model are shown in Fig. 7.

The boxplots show the total weighted maximum workload in each of the 49,967 simulated weeks for each policy. The total weighted maximum workload represents the objective function value (OFV) without the weighted number of ICU blocks. The whiskers are set at 1.5 IQR (interquartile range). The mean total weighted maximum workload which has a strong influence on the workload variability is almost equal for both policies without distinct block types (CMSS: 233.81, NMSS: 233.43) and both policies with distinct block types (CMSSB: 228.95, NMSSB: 228.92). Therefore, both new MSS perform only slightly better than both current MSS. Both MSS with distinct block types perform considerably better than the ones without. Not only is the peak maximum workload within each week, which influences the day-to-day variability, lower for the MSS with distinct block types, but also the variance over all weeks, which reduces the week-to-week variability. The variance of the total weighted maximum workload, and therefore the week-to-week variability of the total weighted maximum workload, is 74.25, 75.32, 62.42, and 64.02 for CMSS, NMSS, CMSSB, and NMSSB, respectively. Both the day-to-day and week-to-week reductions of the total weighted maximum workload result in a constant buffer that could be available for emergency patients and reduces the probability that an elective surgery needs to be canceled due to the capacity limit. In Table 3, a more detailed comparison of the statistical measures is shown for both types of downstream units and both human resource types, namely the ICU and wards, as well as nurses and physicians, respectively, each shown in one of the four quadrants of the table.

**Table 3:** Comparison of statistical measures of total weighted maximum workload for each policy

		Nurse			Physician		
		Median	Mean	Variance	Median	Mean	Variance
ICU	CMSS	21.71	22.56	14.89	10.97	11.12	2.41
	NMSS	21.71	22.53	14.88	10.97	11.12	2.41
	CMSSB	19.54	20.26	10.97	9.56	9.61	1.03
	NMSSB	19.54	20.21	10.83	9.56	9.69	1.09
Ward	CMSS	103.50	103.47	17.38	96.56	96.66	10.39
	NMSS	103.00	103.24	17.32	96.56	96.54	10.13
	CMSSB	103.00	103.14	16.86	96.00	95.95	9.63
	NMSSB	103.00	103.05	17.12	96.00	95.97	9.61

**Fig. 8:**
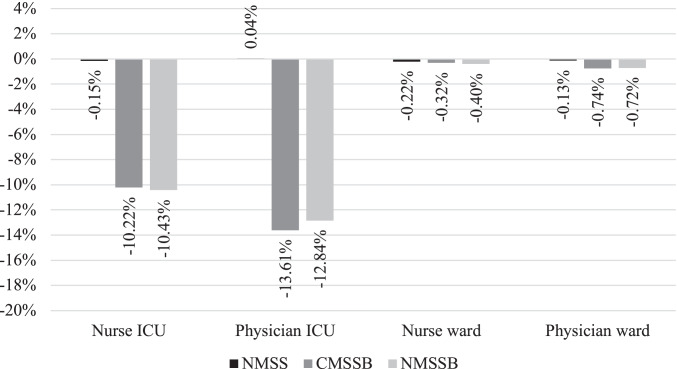
Mean relative change of weighted maximum workload for physicians and nurses in the ICU and wards for NMSS, CMSSB, and NMSSB compared to CMSS

Especially the physicians and nurses in the ICU benefit from a lower total maximum workload when using an MSS with distinct block types with a reduction of the mean maximum workload of up to 10.43% for nurses and up to 13.61% for physicians. Moreover, most of the week-to-week variability reduction is achieved in the ICU with a reduction of the variance of up to 27.22% for nurses and up to 57.03% for physicians. An overview of the resulting relative change in the ICU and wards for both nurses and physicians is shown in Fig. 8. The figure shows the mean relative changes of the total weighted maximum workload compared to the results of the CMSS.

While the distinct block types are most beneficial for reducing the peak workload in the ICU, the maximum workload in the ward stations also sees a small reduction. A reduction of the peak workload is especially beneficial in the ICU due to its service character since it acts as a shared downstream unit used by all surgical specialties in the OT. When comparing CMSSB with NMSSB, there are only small improvements gained from a new MSS. The mean total weighted maximum workload in the ICU for physicians and nurses is 33.68, 29.86, and 29.90 for CMSS, CMSSB, and NMSSB, respectively. This shows that CMSSB can capture all of the potential reduction in the ICU compared to NMSSB. Our approach of maintaining the existing MSS is preferable to hospitals due to the already discussed operational changes associated with the redesign of an MSS. Implementing a new MSS without distinct block types results in a small increase in the total weighted maximum workload in the ICU. As the increase is outweighed by the decrease in the ward stations, there is a small overall reduction. This decrease, however, yields only 7.87% of the reduction that would be possible with NMSSB.

Lastly, we perform an analysis of variance (ANOVA) on the results of the simulation to test for statistical significance of the total maximum workload reduction, as well as the nurse ICU, physician ICU, nurse ward, and physician ward maximum workload reduction of all four policies (CMSS, NMSS, CMSSB, NMSSB). The resulting p-values of all possible policy combinations are then adjusted with the method proposed by Benjamini and Yekutieli [6] to account for multiple hypotheses testing errors. The method by Benjamini and Yekutieli strikes a good balance between unadjusted inference and multiple testing conservatism [4]. The resulting adjusted p-values are shown in Table 4.

**Table 4:** Adjusted p-values from ANOVA post hoc analysis using Benjamini & Yekutieli method

	Total workload	ICU workload		Ward workload	
		Nurse	Physician	Nurse	Physician
CMSS-NMSS	< 0.0001*	0.6791	1.0000	< 0.0001*	< 0.0001*
CMSS-CMSSB	< 0.0001*	< 0.0001*	< 0.0001*	< 0.0001*	< 0.0001*
CMSS-NMSSB	< 0.0001*	< 0.0001*	< 0.0001*	< 0.0001*	< 0.0001*
NMSS-CMSSB	< 0.0001*	< 0.0001*	< 0.0001*	0.0005*	< 0.0001*
NMSS-NMSSB	< 0.0001*	< 0.0001*	< 0.0001*	< 0.0001*	< 0.0001*
CMSSB-NMSSB	1.0000	0.1291	< 0.0001*	0.0025*	1.0000

*: Statistically significant with $\alpha =0.05$

The adjusted p-values from the ANOVA show that the differences between the means of the total maximum workload are all statistically significant except for the CMSSB-NMSSB pair. Looking more closely, the adjusted p-values in this pair show that the differences of means in the nurse ICU maximum workload, as well as the physician ward maximum workload, are not statistically significant with p-values $>0.05$. The results of the CMSS-NMSS pair show that the differences of means are also not statistically significant for both the nurse ICU and the physician ICU maximum workload. These results underline our previous findings and show that with our approach a new MSS is not necessary to level the workload on downstream departments and that a new MSS might not have the desired effect, especially in the ICU.

### Analysis of additional instances

In the last section, we have shown that by introducing two different block types in the MSS of UKA, the workload variability in the ICU can be drastically reduced. However, UKA is one of the largest hospitals in Germany, so it is yet unclear if these results also apply in general, e.g., in smaller hospitals. Therefore, we create multiple instances varying the number of specialties, the size of the OT, and the number of blocks per specialty as well as ICU and ward patient demand in total and per specialty depending on the number of blocks for each specialty. In total, 64 instances are generated. In the first step, 32 base instances are generated. The base instances are constructed as follows: The number of specialties ranges from two to eight, incremented in steps of two. The number of rooms is dependent on the number of specialties and either equal to the number of specialties or twice that number. The total number of ICU patients is set to the number of rooms or twice the number of rooms. The total number of ward patients is generated similarly, only that it is set to five or ten times the number of rooms. In the second step, the 32 base instances are used to generate the final 64 instances with some random parameters: The number of blocks per specialty is generated sequentially starting from the first specialty and is picked from a uniform distribution ranging from zero to half of the remaining blocks that are not assigned yet. However, a minimum of two total specialties is set. The number of ICU and ward patients per specialty is allocated from the total number of ICU and ward patients in relation to the number of blocks per specialty. Lastly, the LOS distributions for the ICU and the ward for each specialty are randomly picked from the eight LOS distributions from UKA. An overview of the parameters for each of the generated instances is shown in Table 5. The CMSS of each instance is generated by evenly distributing the number of blocks per specialty in the planning horizon – except for weekend days – to level the workload of each specialty in the OT as best as possible.

**Table 5:** Overview of generated instances

S	2								4								6								8							
R	2				4				4				8				6				12				8				16			
I	2		4		4		8		4		8		8		16		6		12		12		24		8		16		16		32	
W	10	20	10	20	20	40	20	40	20	40	20	40	40	80	40	80	30	60	30	60	60	120	60	120	40	80	40	80	80	160	80	160
#	1	3	5	7	9	11	13	15	17	19	21	23	25	27	29	31	33	35	37	39	41	43	45	47	49	51	53	55	57	59	61	63
#	2	4	6	8	10	12	14	16	18	20	22	24	26	28	30	32	34	36	38	40	42	44	46	48	50	52	54	56	58	60	62	64

**S:** Number of specialties **R:** Number of OT rooms **I:** Number of total ICU patients **W:** Number of total ward patients **#:** Instance number

Using the generated CMSS of each instance, the three remaining policies NMSS, CMSSB, and NMSSB are applied using the mathematical model and then evaluated using the simulation model. Each instance is evaluated separately by determining the mean maximum workload over all simulated weeks, as well as the standard deviation over all simulated weeks within one policy. The results of NMSS, CMSSB, and NMSSB are then compared to the results of CMSS. The aggregated results are shown in Table 6, showing the mean difference of each policy compared to CMSS showing the mean maximum workload and the mean difference of each policy compared to CMSS showing the standard deviation of the maximum workload. Equal to the results from UKA, it can be seen that NMSS is outperformed by CMSSB and NMSSB, especially in the ICU, not only lowering the mean maximum workload but also lowering the week to week variability measured with the standard deviation. On average, CMSSB reduces the mean maximum workload by 11.06% and 12.40% for nurses and physicians in the ICU, respectively, compared to CMSS. The standard deviation – which represents the week-to-week variability of the maximum workload – could also be decreased by 15.18% and 26.29% for nurses and physicians in the ICU, respectively, on average compared to CMSS. A new MSS with ICU blocks (NMSSB) can reduce the total maximum workload even further by 3.10% compared to the 2.40% reduction when the current MSS of each instance is maintained.

**Table 6:** Mean difference of maximum workload from 64 instances compared to CMSS of each instance using the simulation model

	Policy	Total workload	ICU workload		Ward workload	
			Nurse	Physician	Nurse	Physician
Mean	NMSS	-0.82%	0.20%	0.29%	-0.90%	-1.14%
	CMSSB	-2.40%	-11.06%	-12.40%	-0.36%	-1.01%
	NMSSB	-3.10%	-10.17%	-11.29%	-1.32%	-2.09%
Std.dev	NMSS	0.27%	0.44%	1.34%	-0.88%	-2.14%
	CMSSB	-8.10%	-15.18%	-26.29%	-1.03%	-2.67%
	NMSSB	-7.79%	-13.74%	-23.41%	-1.72%	-3.83%

**Fig. 9:**
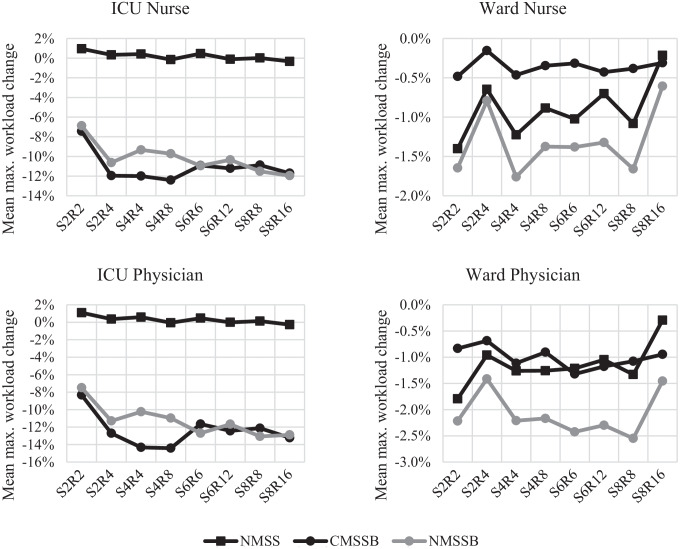
Mean maximum workload change of 64 instances compared to CMSS for all room/specialty combinations – Numbers after S and R represent the number of specialties or rooms

The results of all instances are separated into eight groups, differentiated by the number of rooms and the number of specialties, to assess if these results not only hold on average but also for various OT sizes. In Fig. 9, the mean maximum workload reduction compared to CMSS is shown for NMSS, CMSSB, and NMSSB for nurses and physicians in the ICU and the wards. Similar results as presented in our previous analysis can be observed. The charts show the mean value over all 64 instances divided into eight groups depending on the number of specialties and rooms.

All studied instances show an outperformance of CMSSB and NMSSB compared to NMSS in the ICU independent from the number of rooms and specialties. The reduction of the maximum ICU workload for CMSSB and NMSSB is on a similar level for all instances ranging from small to large, except for the smallest instances with just two rooms and two specialties. The maximum workload on the ward stations shows also no clear trend depending on the size of the instances, here only NMSSB is outperforming CMSS. The results of all instances are shown in Appendix D (online supplements).

## Implementation of ICU quotas at UKA

The presented model was developed in close collaboration with the OT manager of UKA. Due to the convincing improvement potential, the medical director of UKA decided to use the developed model to implement a similar quota system at UKA. Before the implementation of the new model, there were no rules for allocating ICU beds or daily quotas on a tactical level for each specialty, which resulted in major coordination efforts to determine the medical priority of patients relative to the number of available ICU beds on a daily basis. The absence of a controlling mechanism on the tactical level led to a multitude of problems. First and foremost was the impression of an unfair allocation of available ICU beds for all surgical departments since there was no transparent guideline to allocate available ICU beds for elective operations to different specialties. This feeling could also be observed in a questionnaire that was sent out before the new system was implemented, where surgical coordinators of the OT, anesthesia, and all surgical specialties participated. 13 out of 16 participants voted that they were dissatisfied with the current system. Only two participants were neutral, and one participant was somewhat satisfied. Another issue is the high organizational effort that was needed to coordinate the available ICU beds with all involved parties daily, which resulted in an abundance of phone calls between all coordinators. These were symptoms of reactive response management rather than forward-looking active planning. Third, unequal distribution of intensive care patients over the working week was an essential factor for workload distribution into the OT. On some days, this led to a disproportionate rate of delayed or canceled surgeries and a lower overall performance of the OT. Among other things, this was due to a lack of coordination between the surgical specialties and no planning rules.

The attempt to care for as many elective patients as possible in the OT conversely also led to lower ICU capacity reserves for emergency patients. It is essential to be aware that surgical departments have primarily planned intensive care patients according to their needs and restrictions. This can be the availability of surgeons but also device or material requirements.

As transparency was very important to the coordinators of all specialties, the developed model was slightly changed to introduce ICU quotas on a patient level. This allows every specialty to independently plan their ICU patients for the day with the permitted number of elective patients. A modified version of our model was used to continuously support the decision process of determining the daily ICU quotas for each specialty. The model solutions were slightly changed to account for medical and organizational constraints that could not be represented in the model. However, the model was used with fixed decision variables to evaluate the manual changes to the schedules. The final quotas for each specialty were jointly agreed upon by all specialties.

On January 1, 2020, the new system was implemented and is now continuously being evaluated. Part of the evaluation will be additional questionnaires that will be sent out in the future to test the acceptance of the new system amongst the coordinators. Additionally, regarding the acceptance of the system, which is important for the success of the whole project, the LOS in the ICU of elective patients and the number of canceled ICU patients due to bed restrictions will be monitored. For this purpose, a new monitoring system is established to observe the LOS of elective patients in the ICU as well as the fulfillment of the quota system for every specialty. This allows us to check if single specialties comply with the ICU quotas and if a reallocation of the total ICU capacity in the OT is necessary.

Due to the COVID-19 pandemic, the evaluation of data available so far has been impaired because a significantly smaller number of elective patients with ICU treatment needs was operated on. However, in the summer/fall period two months in 2020 were identified where almost no COVID-19 patients were treated in the ICU and the OT could return to regular operations. We use data from September to October 2019 where no quota system was established and compare it to the same period in 2020 where the quota system was established. In the following, we compare the total number of elective surgeries that are transferred to the ICU after surgery with the number of canceled elective ICU surgeries due to bed shortages and we calculate the workload for physicians and nurses in the ICU. Additionally, we present parts of two surveys where 16 coordinators from the OT, the ICU, anesthesia, and from each specialty participated before and after implementation of the ICU quota system.

From September 1, 2019, to October 31, 2019, when ICU quotas had not been established yet, 211 elective surgeries were performed on patients that were transferred to the ICU afterwards. In the same period, 113 surgeries were canceled due to a shortage of ICU beds, resulting in a 34.88% cancellation rate of scheduled elective surgeries of ICU patients. From September 1, 2020, to October 31, 2020, when ICU quotas had been implemented, 208 elective surgeries were performed on ICU patients and only six surgeries had to be canceled due to a bed shortage in the ICU, resulting in a 2.80% cancellation rate of scheduled elective surgeries of ICU patients. This shows that the introduction of ICU quotas had almost no influence on the throughput of ICU patients in the OT, however, the reliability of the planned schedule significantly increased, resulting in less work for coordinators and better predictability for patients.

Since only two months of data were available for comparison, we did not calculate the maximum workload of each week, because only 8 data points would be available. We did calculate the workload of each day and used the standard deviation to measure the workload variability. Looking at the workload for nurses and physicians, the mean weighted workload decreased from 19.54 and 9.77, respectively, in 2019 to 16.45 and 8.23 in 2020. Since the admission rate is nearly equal in both years, the main contributor to the reduction in workload is the reduction of utilized beds followed by a reduction of the average LOS in the ICU. The standard deviation of the weighted workload decreased from 5.77 and 2.89 in 2019 to 5.20 and 2.60 in 2020 for nurses and physicians respectively. However, the coefficient of variation (CoV) slightly increased from 0.30 in 2019 to 0.32 in 2020, resulting in no clear indication if the ICU quotas in the OT have an actual influence on leveling the workload for nurses and physicians in the ICU in practice. An overview of the results from real-world implementation at UKA is shown in Table 7.

**Table 7:** Overview of ICU and OT data before and after implementation of ICU quotas at UKA

	Mean ICU workload		Std. dev. of ICU workload			Total performed ICU surgeries	Canceled ICU surgeries due to bed shortage
	Nurses	Physicians	Nurses	Physicians	CoV		
Without ICU quota system	19.54	9.77	5.77	2.89	0.30	211	113
With ICU quota system	16.45	8.23	5.20	2.60	0.32	208	6

Lastly, when asked about the satisfaction of each system – one without ICU quotas and one with ICU quotas – on a 7-point Likert scale with “very unhappy” at point 1 and “very happy” at point 7, the 16 survey participants were more satisfied with the ICU quota system compared to having no quotas in place with average points of 3.93 compared to 2.25, respectively.

According to the OT manager, the experience of guaranteed ICU beds for patients who were operated on within the quotas led to better discipline when planning these operations, reduced organizational effort, and the feeling of equal treatment in the surgical disciplines. This shows the high acceptance rate of the new system which results from the early involvement of all surgical specialties when adapting the quota system. The quotas also smoothed the demand for ICU beds over the working week. During the COVID-19 pandemic, the new system proved its worth in particular due to its flexibility. Since many patients had to be treated in the ICU because of COVID-19, fewer beds were available for elective surgeries. This reduction could be considered in the dimensioning of daily contingents. This avoided unnecessary cancellations of scheduled ICU surgeries due to bed shortages in the ICU. In addition, daily coordination of the medical prioritization of these patients was introduced at this time. This enables a daily priority list that can be worked through in the designated order regardless of the number of available ICU beds. The quota system has mainly contributed to major transparency in ICU bed allocation.

## Conclusion

In this paper, we present an extension to the MSS using distinct block types for individual downstream units. We built a mathematical model to determine the block type of a specific day and room and thereby create new tactical plans with more information. We use a simulation model to test the workload on an operational level in each downstream unit for physicians and nurses. We show that the weighted maximum workload in the ICU can be reduced by up to 11.22% compared to an MSS without distinct block types using data from UKA in Germany. We also show that we cannot only reduce peak workload within a week but also the week-to-week variability in the ICU using our approach. The main advantage of our approach is possible to capture large fractions of the improvements while maintaining an existing MSS. Compared to generating a new MSS, our approach can realize all of the potential reduction. When compared to a central planning approach, our approach can realize 79.85% of the maximum workload reduction. With our approach, however, specialties keep their autonomy when scheduling patients in their blocks in the OT. Based on the LOS distributions of UKA, we build 64 instances from small to large OTs and few to many specialties combined with a random number of blocks per specialty, ICU patients per specialty, and ward patients per specialty. We show that our approach is not only effective at large OTs but also in smaller hospitals with few rooms and specialties.

Based on our model, a quota system for elective ICU patients in the OT was implemented at the University Hospital Augsburg, one of the largest hospitals in Germany with more than 1,700 beds. The quota system led to an improved organizational structure in the OT and made coordination between surgical specialties easier. The new system showed an almost elimination of cancellations for elective ICU patients in the OT that were canceled due to a bed shortage in the ICU as well as an increased satisfaction of coordinators from the OT, the ICU, and each specialty. However, the reduction of workload variability could not be measured in the observed period.

Our model is mainly focused on downstream units, OT resources are just assumed to be available. Future research could simultaneously focus on the workload within the OT – which we only try to level by limiting the maximum number of daily blocks for each specialty – additionally to the workload on downstream units since many different types of human resources are involved in the OT as well, i.e., physicians, nurses, anesthetists, and anesthesia nurses. Another avenue could be the focus on actual implementation to test if the theoretical potential of workload leveling is transferable to practice, next to other benefits of an ICU quota system that we present in our work.

To summarize our findings, we show that reducing workload peaks on downstream units, increasing employee satisfaction, and reducing organizational costs in the OT is possible without changing the MSS. Using our approach, specialties keep most of their autonomy when scheduling patients in the OT and achieve a majority of the maximum reduction of peak workload that would only be possible with a central planning approach.

## Supplementary Information

Below is the link to the electronic supplementary material.

Supplementary file1 (DOCX 136 KB)
