# Supplementary material for: Balancing control and autonomy in master surgery scheduling: Benefits of ICU quotas for recovery units
Source: Health Care Manag Sci. 2022 Feb 9;25(2):311–32. doi: 10.1007/s10729-021-09588-8 (PMC9165286; doi:10.1007/s10729-021-09588-8)
Supplement: Supplementary file 1 — Supplementary file1 (DOCX 136 KB) [file 10729_2021_9588_MOESM1_ESM.docx]

# Appendix A (Online Supplement)

$D_{c,t}^{\mathrm{ICU}}$ and $D_{c,t,i}^{\mathrm{Ward}}$ are the probabilities convolved onto the planning horizon of $d_{c,q}^{\mathrm{ICU}}$ and $d_{c,q,i}^{\mathrm{Ward}}$ which represent the probabilities that a patient of specialty $c$ stays $q\in Q$ days after surgery in the ICU or ward, respectively. $L_{c,t}^{\mathrm{ICU}}$ and $L_{c,t,i}^{\mathrm{Ward}}$ are the probabilities convolved onto the planning horizon of $l_{c,t}^{\mathrm{ICU}}$ and $l_{c,t,i}^{\mathrm{Ward}}$ which represent the cumulative distribution functions of $d_{c,q}^{\mathrm{ICU}}$ and $d_{c,q,i}^{\mathrm{Ward}}$, stating that a patient stays at least $q\in Q$ days after surgery in the ICU or ward, respectively. Note that $q=0$ is the day of surgery or the transfer from the ICU to the ward. The calculations are shown in (2.1) – (2.4).

| $D_{c,t}^{\mathrm{ICU}}=d_{c,t}^{\mathrm{ICU}}+d_{c,t+\left\vert T \right\vert}^{\mathrm{ICU}}+\ldots+d_{c,t+\left\lfloor\frac{\left\vert Q \right\vert-t-1}{\left\vert T \right\vert} \right\rfloor\left\vert T \right\vert}^{\mathrm{ICU}} \forall c\in C, t\in T$ | (2.1) |
| --- | --- |
| $D_{c,t,i}^{\mathrm{Ward}}=d_{c,t,i}^{\mathrm{Ward}}+d_{c,t+\left\vert T \right\vert,i}^{\mathrm{Ward}}+\ldots+d_{c,t+\left\lfloor\frac{\left\vert Q \right\vert-t-1}{\left\vert T \right\vert} \right\rfloor\left\vert T \right\vert,i}^{\mathrm{Ward}} \forall c\in C, t\in T, i\in I$ | (2.2) |
| $L_{c,t}^{\mathrm{ICU}}=l_{c,t}^{\mathrm{ICU}}+l_{c,t+\left\vert T \right\vert}^{\mathrm{ICU}}+\ldots+l_{c,t+\left\lfloor\frac{\left\vert Q \right\vert-t-1}{\left\vert T \right\vert} \right\rfloor\left\vert T \right\vert}^{\mathrm{ICU}} \forall c\in C, t\in T$ | (2.3) |
| $L_{c,t,i}^{\mathrm{Ward}}=l_{c,t,i}^{\mathrm{Ward}}+l_{c,t+\left\vert T \right\vert,i}^{\mathrm{Ward}}+\ldots+l_{c,t+\left\lfloor\frac{\left\vert Q \right\vert-t-1}{\left\vert T \right\vert} \right\rfloor\left\vert T \right\vert,i}^{\mathrm{Ward}} \forall c\in C, t\in T, i\in I$ | (2.4) |

# Appendix B (Online Supplement)

In the following section, we compare our approach with a completely centralized planning approach where all patients are scheduled by a central planner. Unlike determining an MSS where blocks or surgical groups are generally assigned on a cyclic plan, this model is based on the operational level where individual patients are assigned to rooms and days. For this comparison, we follow the procedure of our solution approach explained in Section 3.2. However, small changes are necessary. Instead of scheduling patients randomly into the MSS as shown in Figure 6, we move this part from the simulation to our mathematical optimization model. The goal of the central planning model is to determine the number of ICU and non-ICU patients for each clinical specialty on each day and each room to minimize the maximum workload in each downstream unit for each human resource type. The presented model is very similar to the one presented in Section 3.2. Instead of using block types and an ICU share which has to meet a target share for each specialty, a set of given ICU and non-ICU patients needs to be scheduled over the planning horizon. The remaining constraints remain unchanged. Additional parameters and decision variables are introduced in the following. Next, we present the central planning model.

| Additional parameters | |
| --- | --- |
| $P_{c.i}^{\mathrm{Patients}}$ | Number of ward ($i=0)$ and ICU ($i=1)$ patients in planning horizon for specialty $c$ |
| $P_{c}^{\mathrm{Max}}$ | Maximum number of patients in a block of specialty $c$ |
| $M$ | Big-M |
| Additional decision variables | |
| $m_{c,r,t}$ | 1 if specialty $c$ has room $r$ on day $t$, 0 otherwise |
| $x_{c,r,t,i}$ | Number of patients of specialty $c$ in room $r$ on day $t$ of type $i$ |

|  | $\min\alpha\cdot\sum_{s\in S} \left( n_{s}^{\mathrm{ICUMax}}+\sum_{c\in C} \left( n_{c,s}^{\mathrm{WardMax}} \right) \right)+\beta\cdot\sum_{s\in S} \left( p_{s}^{\mathrm{ICUMax}}+\sum_{c\in C} \left( p_{c,s}^{\mathrm{WardMax}} \right) \right)$ | (3.1) |
| --- | --- | --- |
| s.t. | |  |
|  | $\sum_{r\in R} \sum_{t\in T} m_{c,r,t}=B_{c}^{\mathrm{Total}} \forall c\in C$ | (3.2) |
|  | $\sum_{r\in R} m_{c,r,t}\leq B_{c}^{\mathrm{Max}} \forall c\in C, t\in T$ | (3.3) |
|  | $m_{c,r,t}\leq0 \forall c\in C, r\in R,t\in O$ | (3.4) |
|  | $\sum_{c\in C} m_{c,r,t}\leq1 \forall r\in R,t\in T$ | (3.5) |
|  | $\sum_{r\in R} \sum_{t\in T} x_{c,r,t,i}=P_{c.i}^{\mathrm{Patients}} \forall c\in C,i\in I$ | (3.6) |
|  | $\sum_{i\in I} x_{c,r,t,i}\leq M\cdot m_{c,r,t} \forall c\in C, r\in R, t\in T$ | (3.7) |
|  | $\sum_{i\in I} x_{c,r,t,i}\leq P_{c}^{\mathrm{Max}} \forall c\in C, r in R, t in T$ | (3.8) |
|  | $\sum_{c\in C} \sum_{r\in R} \sum_{k\in T} L_{c,k}^{\mathrm{ICU}}\cdot x_{c,r,t-k,1}\leq N_{s}^{\mathrm{ICU}}\cdot n_{t,s}^{\mathrm{ICU}} \forall t\in T, s\in S$ | (3.9) |
|  | $n_{t,s}^{\mathrm{ICU}}\leq n_{s}^{\mathrm{ICUMax}} \forall t\in T, s\in S$ | (3.10) |
|  | $\sum_{c\in C} \sum_{r\in R} A_{s}^{\mathrm{ICU}}\cdot x_{c,r,t,1}+\sum_{c\in C} \sum_{r\in R} \sum_{k\in T} R_{t,s}^{\mathrm{ICU}}\cdot L_{c,k}^{\mathrm{ICU}}\cdot x_{c,r,t-k,1}+$  $+\sum_{c\in C} \sum_{r\in R} \sum_{k\in T} D_{s}^{\mathrm{ICU}}\cdot E_{c,t}^{\mathrm{ICU}}\cdot x_{c,r,t-k,1}\leq H_{t,s}\cdot p_{t,s}^{\mathrm{ICU}} \forall t\in T, s\in S$ | (3.11) |
|  | $p_{t,s}^{\mathrm{ICU}}\leq p_{s}^{\mathrm{ICUMax}} \forall t\in T, s\in S$ | (3.12) |
|  | $\sum_{r\in R} \sum_{k\in T} E_{c,t}^{\mathrm{ICU}}\cdot x_{c,r,t-k,1}=y_{c,t,1} \forall c\in C,t\in T$ | (3.13) |
|  | $\sum_{r\in R} x_{c,r,t,0}=y_{c,t,0} \forall c\in C,t\in T$ | (3.14) |
|  | $\sum_{i\in I} \sum_{k\in T} L_{c,k,i}^{\mathrm{Ward}}\cdot y_{c,t-k,i}\leq N_{c,s}^{\mathrm{Ward}}\cdot n_{c,t,s}^{\mathrm{Ward}} \forall c\in C, t\in T, s\in S$ | (3.15) |
|  | $n_{c,t,s}^{\mathrm{Ward}}\leq n_{c,s}^{\mathrm{WardMax}} \forall c\in C, t\in T, s\in S$ | (3.16) |
|  | $\sum_{i\in I} A_{c,s}^{\mathrm{Ward}}\cdot y_{c,t,i}+\sum_{i\in I} \sum_{k\in T} R_{c,t,s}^{\mathrm{Ward}}\cdot L_{c,k,i}^{\mathrm{Ward}}\cdot y_{c,t-k,i}+$  $+\sum_{i\in I} \sum_{k\in T} D_{c,s}^{\mathrm{Ward}}\cdot E_{c,t,i}^{\mathrm{Ward}}\cdot y_{c,t-k,i}\leq H_{t,s}\cdot p_{c,t,s}^{\mathrm{Ward}} \forall c\in C, t\in T, s\in S$ | (3.17) |
|  | $p_{c,t,s}^{\mathrm{Ward}}\leq p_{c,s}^{\mathrm{WardMax}} \forall c\in C, t\in T, s\in S$ | (3.18) |
|  | $m_{c,r,t} \{0,1\}$ | (3.19) |
|  | $b_{c,r,t}^{\mathrm{Share}},n_{t,s}^{\mathrm{ICU}},n_{s}^{\mathrm{ICUMax}},p_{t,s}^{\mathrm{ICU}},p_{s}^{\mathrm{ICUMax}},n_{c,t,s}^{\mathrm{Ward}},n_{c,s}^{\mathrm{WardMax}},p_{c,t,s}^{\mathrm{Ward}},p_{c,s}^{\mathrm{WardMax}},y_{c,t,i}\geq0$ | (3.20) |
|  | $x_{c,r,t,i}\in\mathbb{N}_{0}$ | (3.21) |

The objective function (3.1) minimizes the weighted maximum workload for nurses and physicians in the ICU and the regular wards in each shift. Constraints (3.2) assure that the required number of blocks for each specialty is maintained in the planning horizon. Constraints (3.3) limit the maximum number of daily blocks for each specialty to fulfill the personnel capacity of each specialty. Blocks cannot be assigned to weekend days with Constraints (3.4). The next Constraints (3.5) limit the number of specialties in each room on each day to a maximum of one. Constraints (3.6) are the first constraints different from the other model that assures that a waiting list of ICU and non-ICU patients of each specialty is assigned in the planning horizon. Patients can only be assigned according to the MSS with Constraints (3.7) and are limited to a maximum number of patients in each room by Constraints (3.8). Constraints (3.9) determine the maximum nurse workload in the ICU for each shift and each day by a patient-to-nurse ratio. Constraints (3.10) calculate the highest maximum nurse workload in the ICU in the planning horizon for each shift. In Constraints (3.11) the maximum physician workload in the ICU for each shift and each day is determined by the workload for admissions, daily routine work, and discharges. Constraints (3.12) calculate the highest maximum physician workload in the ICU in the planning horizon for every shift. Constraints (3.13) and (3.14) calculate the inflow of ward patients from the ICU and the OT, respectively. Constraints (3.15) determine the maximum nurse workload in each ward for each shift and day using a patient-to-nurse ratio. The highest maximum nurse workload on each ward for each shift in the planning horizon is computed in Constraints (3.16). The maximum physician workload in each ward for each shift and day is computed in Constraints (3.17) as a workload for the number of admissions, the daily routine work for each patient, and the number of discharges. Constraints (3.18) determine the highest maximum physician workload in each ward for each shift in the planning horizon. Constraints (3.19) - (3.21) define the domain of the decision variables.

In settings where the room assignments must remain unchanged from an existing MSS, Constraints (3.22) are added to the model.

| $m_{c,r,t}=\bar{M}_{c,r,t} \forall c\in C,r\in R, t\in T$ | (3.22) |
| --- | --- |

The final schedule from the mathematical model is then imported into the bottom part of the simulation model shown in Figure 6 labeled “LOS to patient assignment”, and only this part is repeated for every specialty and every simulation run. We evaluate two different policies: A central planning approach with the current MSS (in the following: Central) and a central planning approach with a new MSS (Central New). An overview of the resulting mean relative change for the ICU and wards for both nurses and physicians is shown in Figure 10. The figure shows the mean relative changes of the total weighted maximum workload compared to the results of CMSS.

Figure 10: Mean relative change of total weighted maximum workload for CMSSB and two central planning approaches compared to CMSS

The results indicate that a central planning approach would reduce the total weighted maximum workload even further. However, CMSSB can achieve up to 85.62% of the possible maximum workload reduction for nurses and 71.88% for physicians in the ICU. Therefore, the CMSSB approach realizes up to 79.85% of the reduction of the total weighted maximum workload in the ICU compared to a central planning approach, while maintaining the autonomy of scheduling individual patients at each specialty to some extent. In general wards, the central planning model outperforms our approach. Here, CMSSB captures up to 26.36% of the possible workload reduction compared to a central planning approach. This shows that our approach is most beneficial for reducing the maximum workload in the ICU, which is one of the most expensive resources within a hospital.

# Appendix C (Online Supplement)

In the following section, we perform a sensitivity analysis to show the influence of $\gamma$, the weight associated with the number of ICU blocks, to better understand its influence on the tactical and the operational level. We further explain the reasoning behind choosing $\gamma=0.18$ throughout all studies in our work. To perform this sensitivity analysis, we use CMSSB and follow the solution approach described in Section 3.2. We compare three different KPIs: the number of ICU blocks, the OFV, and the weighted maximum workload, which is the OFV without the weighted number of ICU blocks. The latter two are compared for both the results of the mathematical model and the results of the simulation model. The number of ICU blocks is equal for both models. We incrementally increase the value of $\gamma$ from almost 0 by 0.01 until the number of distinct blocks does not change anymore, i.e., 0.18. We chose a value close to 0 as almost all blocks would be ICU blocks otherwise, resulting in no control over the peak workload on an operational level, as shown in previous sections. The weights for the maximum nurse workload $\alpha$ and the maximum physician workload $\beta$ are set to 2 and 3 respectively, similar to the previous analyses. The results are shown in Figure 11. The number of ICU blocks is shown on the primary vertical axis. The objective function values and the total weighted maximum workload are shown on the secondary vertical axis. The results of the optimization model are marked with squares, the results of the simulation model are marked with rhombi. The OFVs are marked dark grey, the weighted maximum workloads are marked black. As the weight of the number of ICU blocks increases, the number of ICU blocks decreases. Depending on the weight, the number of ICU blocks can be reduced from 22 to 16 $\left( \gamma=0.18 \right)$. The OFV and the total weighted maximum workload are always higher in the simulation evaluation than the optimization results. This is due to the inherent random nature of patient LOS in the simulation model compared to the approximate LOS probabilities in the mathematical model. In both models, the gap between the OFV and the total weighted maximum workload increases because of the increasing weight for the number of ICU blocks, which is part of the OFV. The mathematical model can maintain the total weighted maximum workload at a certain level independently from the number of ICU blocks due to the exact share of ICU patients $b_{c,r,t}$ in each ICU block. However, the continuous nature of the ICU shares in each block $b_{c,r,t}$ that is used for modeling our approach can produce results that cannot be realized in practice and would result in less autonomy for each specialty when scheduling surgeries. This is why we neglect the share in the simulation model and only consider the block type, which we use to evaluate the tactical schedules on an operational level. The strong influence of the total number of ICU blocks on the total weighted maximum workload in downstream units can be seen as well. As the number of ICU blocks decreases, so does the total maximum workload in the simulation model. By increasing the weight for the number of ICU blocks in the mathematical model and thereby decreasing the number of ICU blocks from 22 to 16, the total weighted maximum workload decreases from 231.71 to 228.95 in the simulation model.

Figure 11: Comparison of the number of ICU blocks as well as the weighted maximum workload and OFV of the mathematical model and the simulation model for different $\gamma$-values

In Figure 12, we show the resulting workload for nurses and physicians in each type of downstream unit. A reduction of ICU blocks has a positive effect on the maximum workload in the ICU for both physicians and nurses. Over 70% of the total weighted maximum workload reduction is seen in the ICU. The remaining reduction is distributed over eight different wards, one for each specialty. The maximum workload of the mathematical model remains relatively constant for all values of $\gamma$. For $\gamma$-values larger than 0.18 only the OFV increases. The number of ICU blocks and the total weighted maximum workload does not change any further. To have the maximum benefit on an operational level, which we evaluate with our simulation model, we choose $\gamma=0.18$ throughout all studies in our work.

|  |  |
| --- | --- |
|  |  |
|  | |

Figure 12: Weighted workload in downstream units for physicians and nurses in the mathematical model and the simulation model for different $\gamma$-values

# Appendix D (Online Supplement)

Table 8: Data of created instances

| Instance | Specialties | Rooms | ICU patients | Ward patients | Blocks per specialty | | | | | | | | Number of ICU patients per specialty | | | | | | | | Number of ward patients per specialty | | | | | | | |
| --- | --- | --- | --- | --- | --- | --- | --- | --- | --- | --- | --- | --- | --- | --- | --- | --- | --- | --- | --- | --- | --- | --- | --- | --- | --- | --- | --- | --- |
|  |  |  |  |  | 1 | 2 | 3 | 4 | 5 | 6 | 7 | 8 | 1 | 2 | 3 | 4 | 5 | 6 | 7 | 8 | 1 | 2 | 3 | 4 | 5 | 6 | 7 | 8 |
| 1 | 2 | 2 | 2 | 10 | 5 | 5 |  |  |  |  |  |  | 1 | 1 |  |  |  |  |  |  | 5 | 5 |  |  |  |  |  |  |
| 2 | 2 | 2 | 2 | 10 | 3 | 7 |  |  |  |  |  |  | 1 | 1 |  |  |  |  |  |  | 3 | 7 |  |  |  |  |  |  |
| 3 | 2 | 2 | 2 | 20 | 4 | 6 |  |  |  |  |  |  | 1 | 1 |  |  |  |  |  |  | 8 | 12 |  |  |  |  |  |  |
| 4 | 2 | 2 | 2 | 20 | 3 | 7 |  |  |  |  |  |  | 1 | 1 |  |  |  |  |  |  | 6 | 14 |  |  |  |  |  |  |
| 5 | 2 | 2 | 4 | 10 | 1 | 9 |  |  |  |  |  |  | 0 | 4 |  |  |  |  |  |  | 1 | 9 |  |  |  |  |  |  |
| 6 | 2 | 2 | 4 | 10 | 3 | 7 |  |  |  |  |  |  | 1 | 3 |  |  |  |  |  |  | 3 | 7 |  |  |  |  |  |  |
| 7 | 2 | 2 | 4 | 20 | 5 | 5 |  |  |  |  |  |  | 2 | 2 |  |  |  |  |  |  | 10 | 10 |  |  |  |  |  |  |
| 8 | 2 | 2 | 4 | 20 | 5 | 5 |  |  |  |  |  |  | 2 | 2 |  |  |  |  |  |  | 10 | 10 |  |  |  |  |  |  |
| 9 | 2 | 4 | 2 | 10 | 4 | 16 |  |  |  |  |  |  | 1 | 3 |  |  |  |  |  |  | 4 | 16 |  |  |  |  |  |  |
| 10 | 2 | 4 | 2 | 10 | 2 | 18 |  |  |  |  |  |  | 0 | 4 |  |  |  |  |  |  | 2 | 18 |  |  |  |  |  |  |
| 11 | 2 | 4 | 2 | 20 | 3 | 17 |  |  |  |  |  |  | 1 | 3 |  |  |  |  |  |  | 6 | 34 |  |  |  |  |  |  |
| 12 | 2 | 4 | 2 | 20 | 5 | 15 |  |  |  |  |  |  | 1 | 3 |  |  |  |  |  |  | 10 | 30 |  |  |  |  |  |  |
| 13 | 2 | 4 | 4 | 10 | 10 | 10 |  |  |  |  |  |  | 4 | 4 |  |  |  |  |  |  | 10 | 10 |  |  |  |  |  |  |
| 14 | 2 | 4 | 4 | 10 | 4 | 16 |  |  |  |  |  |  | 2 | 6 |  |  |  |  |  |  | 4 | 16 |  |  |  |  |  |  |
| 15 | 2 | 4 | 4 | 20 | 6 | 14 |  |  |  |  |  |  | 2 | 6 |  |  |  |  |  |  | 12 | 28 |  |  |  |  |  |  |
| 16 | 2 | 4 | 4 | 20 | 7 | 13 |  |  |  |  |  |  | 3 | 5 |  |  |  |  |  |  | 14 | 26 |  |  |  |  |  |  |
| 17 | 4 | 4 | 4 | 20 | 7 | 6 | 1 | 6 |  |  |  |  | 1 | 1 | 0 | 2 |  |  |  |  | 7 | 6 | 1 | 6 |  |  |  |  |
| 18 | 4 | 4 | 4 | 20 | 4 | 4 | 4 | 8 |  |  |  |  | 1 | 1 | 1 | 1 |  |  |  |  | 4 | 4 | 4 | 8 |  |  |  |  |
| 19 | 4 | 4 | 4 | 40 | 2 | 4 | 7 | 7 |  |  |  |  | 0 | 1 | 1 | 2 |  |  |  |  | 4 | 8 | 14 | 14 |  |  |  |  |
| 20 | 4 | 4 | 4 | 40 | 5 | 3 | 3 | 9 |  |  |  |  | 1 | 1 | 1 | 1 |  |  |  |  | 10 | 6 | 6 | 18 |  |  |  |  |
| 21 | 4 | 4 | 8 | 20 | 3 | 7 | 0 | 10 |  |  |  |  | 1 | 3 | 0 | 4 |  |  |  |  | 3 | 7 | 0 | 10 |  |  |  |  |
| 22 | 4 | 4 | 8 | 20 | 8 | 6 | 1 | 5 |  |  |  |  | 3 | 2 | 0 | 3 |  |  |  |  | 8 | 6 | 1 | 5 |  |  |  |  |
| 23 | 4 | 4 | 8 | 40 | 10 | 2 | 4 | 4 |  |  |  |  | 4 | 1 | 2 | 1 |  |  |  |  | 20 | 4 | 8 | 8 |  |  |  |  |
| 24 | 4 | 4 | 8 | 40 | 10 | 5 | 0 | 5 |  |  |  |  | 4 | 2 | 0 | 2 |  |  |  |  | 20 | 10 | 0 | 10 |  |  |  |  |
| 25 | 4 | 8 | 4 | 20 | 14 | 3 | 5 | 18 |  |  |  |  | 3 | 1 | 1 | 3 |  |  |  |  | 14 | 3 | 5 | 18 |  |  |  |  |
| 26 | 4 | 8 | 4 | 20 | 12 | 11 | 8 | 9 |  |  |  |  | 2 | 2 | 2 | 2 |  |  |  |  | 12 | 11 | 8 | 9 |  |  |  |  |
| 27 | 4 | 8 | 4 | 40 | 11 | 14 | 6 | 9 |  |  |  |  | 2 | 3 | 1 | 2 |  |  |  |  | 22 | 28 | 12 | 18 |  |  |  |  |
| 28 | 4 | 8 | 4 | 40 | 8 | 2 | 2 | 28 |  |  |  |  | 2 | 0 | 0 | 6 |  |  |  |  | 16 | 4 | 4 | 56 |  |  |  |  |
| 29 | 4 | 8 | 8 | 20 | 19 | 5 | 5 | 11 |  |  |  |  | 8 | 2 | 2 | 4 |  |  |  |  | 19 | 5 | 5 | 11 |  |  |  |  |
| 30 | 4 | 8 | 8 | 20 | 3 | 2 | 3 | 32 |  |  |  |  | 1 | 1 | 1 | 13 |  |  |  |  | 3 | 2 | 3 | 32 |  |  |  |  |
| 31 | 4 | 8 | 8 | 40 | 19 | 8 | 2 | 11 |  |  |  |  | 8 | 3 | 1 | 4 |  |  |  |  | 38 | 16 | 4 | 22 |  |  |  |  |
| 32 | 4 | 8 | 8 | 40 | 8 | 1 | 11 | 20 |  |  |  |  | 3 | 0 | 4 | 9 |  |  |  |  | 16 | 2 | 22 | 40 |  |  |  |  |
| 33 | 6 | 6 | 6 | 30 | 1 | 10 | 2 | 1 | 8 | 8 |  |  | 0 | 2 | 0 | 0 | 2 | 2 |  |  | 1 | 10 | 2 | 1 | 8 | 8 |  |  |
| 34 | 6 | 6 | 6 | 30 | 9 | 1 | 3 | 6 | 3 | 8 |  |  | 2 | 0 | 1 | 1 | 1 | 1 |  |  | 9 | 1 | 3 | 6 | 3 | 8 |  |  |
| 35 | 6 | 6 | 6 | 60 | 2 | 14 | 1 | 6 | 1 | 6 |  |  | 0 | 3 | 0 | 1 | 0 | 2 |  |  | 4 | 28 | 2 | 12 | 2 | 12 |  |  |
| 36 | 6 | 6 | 6 | 60 | 7 | 0 | 8 | 7 | 4 | 4 |  |  | 1 | 0 | 2 | 1 | 1 | 1 |  |  | 14 | 0 | 16 | 14 | 8 | 8 |  |  |
| 37 | 6 | 6 | 12 | 30 | 1 | 12 | 1 | 4 | 1 | 11 |  |  | 0 | 5 | 0 | 2 | 0 | 5 |  |  | 1 | 12 | 1 | 4 | 1 | 11 |  |  |
| 38 | 6 | 6 | 12 | 30 | 14 | 7 | 4 | 1 | 1 | 3 |  |  | 6 | 3 | 2 | 0 | 0 | 1 |  |  | 14 | 7 | 4 | 1 | 1 | 3 |  |  |
| 39 | 6 | 6 | 12 | 60 | 9 | 5 | 1 | 6 | 0 | 9 |  |  | 4 | 2 | 0 | 2 | 0 | 4 |  |  | 18 | 10 | 2 | 12 | 0 | 18 |  |  |
| 40 | 6 | 6 | 12 | 60 | 14 | 6 | 2 | 4 | 0 | 4 |  |  | 6 | 2 | 1 | 2 | 0 | 1 |  |  | 28 | 12 | 4 | 8 | 0 | 8 |  |  |
| 41 | 6 | 12 | 6 | 30 | 28 | 8 | 10 | 6 | 3 | 5 |  |  | 6 | 2 | 2 | 1 | 1 | 0 |  |  | 28 | 8 | 10 | 6 | 3 | 5 |  |  |
| 42 | 6 | 12 | 6 | 30 | 12 | 0 | 19 | 6 | 8 | 15 |  |  | 2 | 0 | 4 | 1 | 2 | 3 |  |  | 12 | 0 | 19 | 6 | 8 | 15 |  |  |
| 43 | 6 | 12 | 6 | 60 | 22 | 11 | 11 | 7 | 3 | 6 |  |  | 4 | 2 | 2 | 1 | 1 | 2 |  |  | 44 | 22 | 22 | 14 | 6 | 12 |  |  |
| 44 | 6 | 12 | 6 | 60 | 13 | 19 | 4 | 9 | 4 | 11 |  |  | 3 | 4 | 1 | 2 | 1 | 1 |  |  | 26 | 38 | 8 | 18 | 8 | 22 |  |  |
| 45 | 6 | 12 | 12 | 30 | 19 | 12 | 3 | 2 | 10 | 14 |  |  | 8 | 5 | 1 | 1 | 4 | 5 |  |  | 19 | 12 | 3 | 2 | 10 | 14 |  |  |
| 46 | 6 | 12 | 12 | 30 | 30 | 7 | 7 | 7 | 0 | 9 |  |  | 12 | 3 | 3 | 3 | 0 | 3 |  |  | 30 | 7 | 7 | 7 | 0 | 9 |  |  |
| 47 | 6 | 12 | 12 | 60 | 24 | 13 | 6 | 2 | 0 | 15 |  |  | 10 | 5 | 2 | 1 | 0 | 6 |  |  | 48 | 26 | 12 | 4 | 0 | 30 |  |  |
| 48 | 6 | 12 | 12 | 60 | 24 | 10 | 1 | 12 | 6 | 7 |  |  | 10 | 4 | 0 | 5 | 2 | 3 |  |  | 48 | 20 | 2 | 24 | 12 | 14 |  |  |
| 49 | 8 | 8 | 8 | 40 | 12 | 8 | 0 | 5 | 7 | 0 | 4 | 4 | 2 | 2 | 0 | 1 | 1 | 0 | 1 | 1 | 12 | 8 | 0 | 5 | 7 | 0 | 4 | 4 |
| 50 | 8 | 8 | 8 | 40 | 3 | 4 | 4 | 0 | 0 | 12 | 1 | 16 | 1 | 1 | 1 | 0 | 0 | 2 | 0 | 3 | 3 | 4 | 4 | 0 | 0 | 12 | 1 | 16 |
| 51 | 8 | 8 | 8 | 80 | 14 | 8 | 0 | 0 | 4 | 4 | 4 | 6 | 3 | 2 | 0 | 0 | 1 | 1 | 1 | 0 | 28 | 16 | 0 | 0 | 8 | 8 | 8 | 12 |
| 52 | 8 | 8 | 8 | 80 | 18 | 6 | 5 | 0 | 2 | 1 | 4 | 4 | 4 | 1 | 1 | 0 | 0 | 0 | 1 | 1 | 36 | 12 | 10 | 0 | 4 | 2 | 8 | 8 |
| 53 | 8 | 8 | 16 | 40 | 7 | 9 | 4 | 10 | 1 | 2 | 0 | 7 | 3 | 4 | 2 | 4 | 0 | 1 | 0 | 2 | 7 | 9 | 4 | 10 | 1 | 2 | 0 | 7 |
| 54 | 8 | 8 | 16 | 40 | 16 | 11 | 2 | 4 | 0 | 2 | 1 | 4 | 6 | 4 | 1 | 2 | 0 | 1 | 0 | 2 | 16 | 11 | 2 | 4 | 0 | 2 | 1 | 4 |
| 55 | 8 | 8 | 16 | 80 | 9 | 12 | 8 | 0 | 3 | 2 | 1 | 5 | 4 | 5 | 3 | 0 | 1 | 1 | 0 | 2 | 18 | 24 | 16 | 0 | 6 | 4 | 2 | 10 |
| 56 | 8 | 8 | 16 | 80 | 12 | 4 | 10 | 5 | 3 | 3 | 0 | 3 | 5 | 2 | 4 | 2 | 1 | 1 | 0 | 1 | 24 | 8 | 20 | 10 | 6 | 6 | 0 | 6 |
| 57 | 8 | 16 | 8 | 40 | 32 | 16 | 14 | 7 | 4 | 2 | 0 | 5 | 6 | 3 | 3 | 1 | 1 | 0 | 0 | 2 | 32 | 16 | 14 | 7 | 4 | 2 | 0 | 5 |
| 58 | 8 | 16 | 8 | 40 | 22 | 24 | 7 | 3 | 3 | 10 | 5 | 6 | 4 | 5 | 1 | 1 | 1 | 2 | 1 | 1 | 22 | 24 | 7 | 3 | 3 | 10 | 5 | 6 |
| 59 | 8 | 16 | 8 | 80 | 26 | 19 | 8 | 0 | 13 | 4 | 0 | 10 | 5 | 4 | 2 | 0 | 3 | 1 | 0 | 1 | 52 | 38 | 16 | 0 | 26 | 8 | 0 | 20 |
| 60 | 8 | 16 | 8 | 80 | 18 | 19 | 15 | 11 | 7 | 2 | 0 | 8 | 4 | 4 | 3 | 2 | 1 | 0 | 0 | 2 | 36 | 38 | 30 | 22 | 14 | 4 | 0 | 16 |
| 61 | 8 | 16 | 16 | 40 | 9 | 33 | 1 | 14 | 4 | 3 | 7 | 9 | 4 | 13 | 0 | 6 | 2 | 1 | 3 | 3 | 9 | 33 | 1 | 14 | 4 | 3 | 7 | 9 |
| 62 | 8 | 16 | 16 | 40 | 14 | 9 | 17 | 4 | 14 | 7 | 3 | 12 | 6 | 4 | 7 | 2 | 6 | 3 | 1 | 3 | 14 | 9 | 17 | 4 | 14 | 7 | 3 | 12 |
| 63 | 8 | 16 | 16 | 80 | 31 | 13 | 9 | 13 | 1 | 4 | 2 | 7 | 12 | 5 | 4 | 5 | 0 | 2 | 1 | 3 | 62 | 26 | 18 | 26 | 2 | 8 | 4 | 14 |
| 64 | 8 | 16 | 16 | 80 | 16 | 30 | 3 | 10 | 4 | 7 | 1 | 9 | 6 | 12 | 1 | 4 | 2 | 3 | 0 | 4 | 32 | 60 | 6 | 20 | 8 | 14 | 2 | 18 |

Table 9: Results of 64 instances – Mean difference of maximum workload compared to CMSS from the simulation model

| Instance | Total max. workload | | | Nurse ICU max. workload | | | Physician ICU max. workload | | | Nurse ward max. workload | | | Physician ward max. workload | | |
| --- | --- | --- | --- | --- | --- | --- | --- | --- | --- | --- | --- | --- | --- | --- | --- |
|  | NMSS | CMSSB | NMSSB | NMSS | CMSSB | NMSSB | NMSS | CMSSB | NMSSB | NMSS | CMSSB | NMSSB | NMSS | CMSSB | NMSSB |
| 1 | 0,13% | -1,97% | -1,84% | -0,09% | -9,39% | -9,27% | -0,17% | -9,94% | -9,80% | 0,23% | 0,01% | 0,15% | 0,16% | -0,11% | 0,02% |
| 2 | -2,41% | -3,00% | -4,36% | 3,38% | -7,66% | -3,15% | 3,31% | -7,82% | -3,29% | -3,29% | -1,74% | -3,94% | -4,82% | -1,56% | -5,43% |
| 3 | -0,47% | -1,41% | -2,22% | 1,50% | -7,98% | -8,14% | 1,79% | -8,50% | -8,54% | -0,78% | -0,15% | -1,11% | -0,75% | -0,73% | -1,59% |
| 4 | -4,19% | -2,07% | -5,46% | 2,74% | -6,63% | -5,78% | 2,65% | -7,78% | -6,00% | -4,93% | -0,28% | -4,94% | -5,60% | -2,26% | -5,84% |
| 5 | -1,01% | -2,23% | -3,18% | -0,35% | -8,44% | -8,74% | -0,39% | -9,28% | -9,52% | -0,93% | 0,01% | -0,99% | -1,51% | -0,22% | -1,57% |
| 6 | -0,76% | -1,29% | -2,25% | 0,81% | -2,28% | -2,78% | 1,70% | -4,51% | -4,07% | -1,32% | -0,53% | -1,34% | -1,69% | -0,68% | -2,42% |
| 7 | 0,07% | -1,33% | -1,25% | -0,33% | -4,40% | -4,51% | -0,14% | -5,56% | -5,70% | 0,18% | -0,35% | -0,22% | 0,14% | -0,45% | -0,31% |
| 8 | -0,28% | -3,17% | -3,10% | -0,06% | -12,73% | -12,67% | 0,00% | -13,22% | -13,03% | -0,38% | -0,84% | -0,78% | -0,29% | -0,66% | -0,59% |
| 9 | -0,74% | -3,18% | -3,33% | 0,28% | -14,01% | -13,48% | 0,16% | -14,76% | -14,01% | -0,75% | -0,31% | -1,05% | -1,17% | -1,44% | -1,29% |
| 10 | -1,49% | -3,04% | -4,92% | -0,20% | -13,14% | -13,12% | -0,09% | -13,75% | -13,79% | -1,57% | -0,03% | -2,14% | -2,14% | -0,34% | -3,03% |
| 11 | -0,51% | -1,39% | -1,76% | 1,08% | -12,35% | -11,74% | 1,12% | -12,68% | -11,34% | -0,58% | 0,17% | -0,43% | -0,87% | 0,01% | -0,46% |
| 12 | 0,02% | -1,11% | -1,16% | 0,12% | -6,89% | -6,84% | 0,15% | -8,54% | -8,46% | 0,00% | -0,11% | -0,17% | 0,00% | -0,09% | -0,16% |
| 13 | 0,14% | -1,10% | -0,98% | -0,09% | -5,98% | -5,92% | -0,10% | -6,20% | -6,17% | 0,22% | 0,41% | 0,56% | 0,19% | 0,18% | 0,30% |
| 14 | -0,68% | -2,27% | -3,79% | 0,35% | -10,17% | -12,13% | 0,43% | -10,36% | -12,50% | -0,75% | 0,04% | -0,89% | -1,25% | -0,08% | -1,94% |
| 15 | -0,59% | -2,19% | -3,10% | 0,10% | -9,61% | -12,77% | 0,32% | -11,37% | -14,02% | -0,56% | -0,29% | -0,77% | -0,93% | -1,29% | -1,90% |
| 16 | -0,97% | -5,39% | -3,33% | 1,02% | -23,49% | -9,02% | 0,87% | -23,98% | -10,09% | -1,20% | -1,11% | -1,45% | -1,52% | -2,43% | -2,84% |
| 17 | -0,98% | -2,26% | -3,30% | 0,47% | -12,30% | -8,29% | 0,39% | -14,41% | -8,93% | -0,99% | 0,44% | -1,71% | -1,59% | -0,24% | -2,60% |
| 18 | -1,08% | -3,21% | -3,20% | 2,05% | -11,45% | -8,63% | 2,71% | -15,71% | -8,74% | -1,89% | -0,24% | -1,55% | -1,81% | -1,78% | -2,36% |
| 19 | -1,60% | -3,59% | -2,29% | 0,57% | -17,20% | -2,85% | 0,59% | -19,98% | -3,83% | -1,97% | -0,78% | -2,13% | -1,80% | -2,61% | -2,22% |
| 20 | -1,40% | -1,15% | -3,11% | -0,29% | -9,56% | -10,10% | 0,52% | -13,57% | -10,82% | -1,59% | -0,10% | -2,13% | -1,53% | 0,04% | -2,44% |
| 21 | -0,80% | -3,99% | -5,09% | -0,42% | -10,21% | -12,97% | -0,22% | -11,52% | -13,33% | -0,87% | -1,48% | -2,43% | -1,02% | -2,47% | -2,89% |
| 22 | -0,76% | -2,77% | -2,97% | 0,12% | -10,20% | -7,70% | 0,23% | -10,59% | -8,55% | -0,89% | -0,59% | -1,08% | -1,17% | -0,63% | -2,03% |
| 23 | -0,99% | -2,07% | -3,64% | 0,70% | -10,52% | -9,69% | 0,38% | -12,65% | -11,63% | -1,50% | -0,32% | -2,42% | -1,06% | -0,37% | -2,32% |
| 24 | -0,08% | -2,96% | -2,92% | 0,10% | -14,51% | -14,41% | 0,08% | -16,15% | -16,12% | -0,12% | -0,66% | -0,62% | -0,10% | -0,86% | -0,83% |
| 25 | -0,48% | -2,15% | -3,04% | -0,74% | -9,86% | -11,00% | -0,34% | -11,41% | -12,47% | -0,01% | -0,36% | -0,83% | -0,92% | -0,49% | -1,74% |
| 26 | -0,39% | -2,45% | -2,66% | -0,12% | -12,68% | -10,35% | 0,02% | -14,11% | -11,52% | -0,31% | -0,29% | -0,66% | -0,59% | -0,56% | -1,62% |
| 27 | -0,89% | -2,17% | -2,07% | 0,19% | -15,48% | -5,19% | 0,14% | -19,68% | -5,54% | -0,94% | -0,36% | -1,39% | -1,08% | -0,62% | -2,05% |
| 28 | -3,32% | -1,77% | -4,71% | -0,54% | -11,58% | -12,30% | -0,39% | -12,62% | -12,71% | -3,94% | -0,50% | -3,86% | -3,33% | -0,73% | -3,79% |
| 29 | -0,36% | -2,86% | -2,99% | -0,38% | -12,50% | -10,87% | -0,40% | -13,75% | -12,07% | -0,26% | -0,17% | -0,65% | -0,45% | -0,62% | -1,28% |
| 30 | -0,85% | -2,68% | -3,61% | 0,15% | -8,90% | -9,09% | 0,35% | -12,81% | -11,90% | -0,75% | 0,05% | -1,04% | -1,65% | -0,83% | -2,28% |
| 31 | -0,50% | -3,01% | -3,53% | -0,05% | -14,41% | -12,01% | 0,06% | -15,49% | -12,57% | -0,12% | -0,40% | -1,39% | -1,07% | -1,51% | -2,65% |
| 32 | -0,68% | -3,52% | -2,58% | 0,26% | -13,83% | -6,98% | 0,10% | -15,42% | -8,96% | -0,76% | -0,73% | -1,17% | -0,96% | -1,87% | -1,93% |
| 33 | -0,90% | -1,66% | -2,74% | -0,85% | -7,91% | -8,73% | -0,89% | -8,26% | -9,34% | -0,89% | -0,16% | -1,15% | -0,92% | -0,59% | -1,82% |
| 34 | -1,28% | -2,44% | -4,29% | 0,56% | -9,52% | -12,76% | 1,34% | -9,55% | -14,43% | -1,48% | -0,45% | -1,86% | -1,93% | -1,60% | -3,10% |
| 35 | -1,02% | -1,85% | -3,18% | 0,84% | -12,20% | -10,97% | 0,72% | -12,95% | -13,54% | -1,02% | -0,08% | -1,51% | -1,46% | -1,17% | -2,86% |
| 36 | -1,38% | -2,20% | -4,73% | 0,33% | -13,68% | -20,91% | 0,48% | -14,17% | -22,83% | -1,61% | -0,48% | -2,28% | -1,58% | -1,16% | -3,19% |
| 37 | -0,40% | -2,77% | -2,80% | 0,21% | -10,19% | -6,49% | 0,06% | -10,66% | -9,29% | -0,44% | -0,26% | -0,99% | -0,68% | -0,92% | -1,95% |
| 38 | -0,36% | -3,48% | -3,21% | 0,92% | -10,99% | -8,31% | 0,56% | -12,33% | -10,59% | -0,52% | -0,39% | -0,78% | -0,89% | -1,96% | -2,24% |
| 39 | -0,71% | -2,49% | -2,42% | 0,23% | -10,13% | -9,38% | 0,48% | -11,04% | -9,68% | -0,84% | -0,43% | -0,78% | -0,97% | -1,60% | -1,44% |
| 40 | -0,89% | -3,00% | -3,66% | 1,42% | -12,75% | -10,08% | 0,96% | -14,22% | -11,92% | -1,38% | -0,27% | -1,70% | -1,27% | -1,57% | -2,77% |
| 41 | -0,72% | -3,07% | -2,67% | -0,42% | -13,60% | -8,96% | -0,24% | -14,88% | -10,29% | -0,63% | -0,57% | -0,93% | -0,96% | -1,45% | -1,90% |
| 42 | -0,43% | -2,21% | -2,63% | -0,21% | -10,83% | -9,58% | -0,15% | -12,40% | -11,60% | -0,37% | -0,22% | -0,93% | -0,59% | -0,98% | -1,65% |
| 43 | -1,65% | -2,25% | -3,83% | 0,56% | -11,05% | -10,24% | 0,91% | -12,59% | -11,49% | -1,68% | -0,73% | -2,60% | -2,14% | -1,80% | -3,61% |
| 44 | -0,92% | -1,64% | -2,75% | 0,25% | -10,12% | -13,06% | 0,29% | -12,00% | -14,20% | -0,97% | -0,32% | -1,28% | -1,13% | -1,04% | -1,97% |
| 45 | -0,54% | -2,49% | -3,60% | -0,24% | -9,06% | -10,59% | -0,23% | -9,65% | -11,53% | -0,32% | -0,64% | -1,22% | -0,92% | -1,13% | -2,51% |
| 46 | -0,37% | -1,87% | -2,56% | -0,32% | -6,49% | -7,60% | -0,20% | -7,63% | -8,71% | -0,29% | -0,39% | -0,77% | -0,50% | -0,84% | -1,62% |
| 47 | -0,36% | -2,45% | -2,97% | -0,50% | -15,42% | -12,13% | -0,44% | -16,56% | -13,68% | -0,20% | -0,07% | -1,03% | -0,50% | -0,90% | -2,06% |
| 48 | -1,21% | -2,62% | -3,63% | -0,01% | -13,14% | -10,63% | -0,03% | -13,72% | -11,87% | -1,16% | -0,49% | -1,82% | -1,65% | -1,29% | -3,06% |
| 49 | -0,79% | -2,27% | -3,30% | -0,56% | -11,94% | -11,58% | -0,49% | -13,25% | -13,67% | -0,56% | -0,21% | -1,37% | -1,13% | -0,54% | -1,87% |
| 50 | -0,54% | -2,20% | -3,34% | -0,36% | -10,94% | -13,02% | -0,28% | -12,76% | -15,01% | -0,45% | 0,00% | -0,86% | -0,73% | -0,77% | -1,79% |
| 51 | -1,23% | -1,83% | -3,55% | 0,57% | -12,77% | -12,98% | 0,90% | -14,85% | -15,41% | -1,33% | -0,25% | -1,80% | -1,58% | -0,76% | -2,98% |
| 52 | -1,02% | -1,75% | -3,58% | -0,09% | -6,38% | -15,75% | -0,12% | -8,01% | -18,75% | -1,15% | -0,77% | -1,58% | -1,11% | -1,50% | -2,42% |
| 53 | -0,55% | -3,04% | -2,84% | 0,28% | -12,03% | -8,09% | 0,38% | -13,05% | -8,30% | -0,46% | -0,19% | -0,90% | -1,10% | -1,03% | -2,04% |
| 54 | -0,91% | -3,00% | -3,33% | 0,01% | -10,78% | -8,96% | 0,05% | -12,04% | -9,92% | -0,99% | -0,60% | -1,40% | -1,29% | -1,53% | -2,45% |
| 55 | -2,34% | -3,18% | -5,37% | -0,33% | -13,44% | -12,12% | -0,07% | -14,97% | -13,42% | -2,66% | -0,83% | -3,54% | -2,76% | -1,74% | -4,68% |
| 56 | -0,77% | -1,62% | -3,10% | 0,60% | -8,79% | -9,61% | 0,67% | -8,09% | -10,03% | -1,05% | -0,21% | -1,83% | -0,95% | -0,74% | -2,17% |
| 57 | -0,37% | -2,12% | -2,97% | -0,01% | -10,13% | -11,58% | -0,04% | -11,10% | -12,81% | -0,37% | -0,44% | -1,04% | -0,50% | -0,66% | -1,50% |
| 58 | 0,04% | -2,61% | -2,68% | -0,27% | -11,94% | -11,53% | -0,13% | -13,75% | -13,43% | -0,05% | -0,54% | -0,67% | 0,24% | -1,00% | -1,17% |
| 59 | -0,10% | -2,05% | -2,11% | -0,01% | -18,88% | -17,32% | -0,05% | -19,68% | -18,35% | -0,15% | 0,06% | -0,20% | -0,07% | -0,77% | -0,93% |
| 60 | -0,86% | -1,81% | -2,65% | -0,38% | -12,01% | -11,19% | -0,29% | -15,03% | -12,72% | -0,70% | -0,27% | -1,27% | -1,13% | -1,05% | -2,18% |
| 61 | -0,45% | -2,31% | -2,49% | -1,08% | -10,88% | -10,90% | -1,03% | -11,89% | -10,93% | -0,17% | 0,17% | -0,09% | -0,46% | -0,75% | -1,06% |
| 62 | 0,04% | -2,49% | -2,59% | -0,46% | -8,81% | -9,61% | -0,42% | -10,14% | -9,46% | 0,16% | -0,61% | -0,55% | 0,13% | -1,19% | -1,36% |
| 63 | -0,29% | -1,90% | -2,67% | -0,18% | -9,47% | -11,49% | -0,12% | -10,03% | -12,45% | -0,27% | -0,22% | -0,52% | -0,35% | -1,03% | -1,83% |
| 64 | -0,19% | -2,44% | -2,58% | -0,25% | -11,53% | -12,04% | -0,13% | -14,14% | -12,97% | -0,17% | -0,63% | -0,52% | -0,21% | -1,11% | -1,60% |
